# Standardization of the FAO/IAEA Flight Test for Quality Control of Sterile Mosquitoes

**DOI:** 10.3389/fbioe.2022.876675

**Published:** 2022-07-18

**Authors:** Hamidou Maïga, Deng Lu, Wadaka Mamai, Nanwintoum Séverin Bimbilé Somda, Thomas Wallner, Mame Thierno Bakhoum, Odet Bueno Masso, Claudia Martina, Simran Singh Kotla, Hanano Yamada, Gustavo Salvador Herranz, Rafael Argiles Herrero, Chee Seng Chong, Cheong Huat Tan, Jeremy Bouyer

**Affiliations:** ^1^ Insect Pest Control Laboratory, Joint FAO/IAEA Centre of Nuclear Techniques in Food and Agriculture, Department of Nuclear Sciences and Applications, IAEA Laboratories, Seibersdorf, Austria; ^2^ Institut de Recherche en Sciences de la Santé/Direction Régionale de l’Ouest (IRSS-DRO), Bobo-Dioulasso, Burkina Faso; ^3^ Environmental Health Institute, National Environnent Agency, Singapore, Singapore; ^4^ Institut de Recherche Agricole pour le Développement (IRAD), Yaoundé-Messa, Cameroon; ^5^ Unité de Formation et de Recherche en Sciences et Technologies (UFR/ST), Université Norbert ZONGO (UNZ), Koudougou, Burkina Faso; ^6^ Institut Sénégalais de Recherches Agricoles (ISRA), Dakar, Senegal; ^7^ Technical School of Design, Architecture and Engineering, University CEU Cardenal Herrera, Valencia, Spain

**Keywords:** mass-rearing, sterile insect technique, *Aedes aegypti*, *Aedes albopictus*, age, cost, color

## Abstract

Successful implementation of the sterile insect technique (SIT) against *Aedes aegypti and Aedes albopictus* relies on maintaining a consistent release of high-quality sterile males. Affordable, rapid, practical quality control tools based on the male’s flight ability (ability to escape from a flight device) may contribute to meeting this requirement. Therefore, this study aims to standardize the use of the original FAO/IAEA rapid quality control flight test device (FTD) (version 1.0), while improving handling conditions and reducing the device’s overall cost by assessing factors that could impact the subsequent flight ability of *Aedes* mosquitoes. The new FTD (version 1.1) is easier to use. The most important factors affecting escape rates were found to be tube color (or “shade”), the combined use of a lure and fan, mosquito species, and mosquito age and density (25; 50; 75; 100 males). Other factors measured but found to be less important were the duration of the test (30, 60, 90, 120 min), fan speed (normal 3000 rpm vs. high 6000 rpm), and mosquito strain origin. In addition, a cheaper version of the FTD (version 2.0) that holds eight individual tubes instead of 40 was designed and successfully validated against the new FTD (version 1.1). It was sensitive enough to distinguish between the effects of cold stress and high irradiation dose. Therefore, the eight-tube FTD may be used to assess *Aedes’* flight ability. This study demonstrated that the new designs (versions 1.1 and 2.0) of the FTD could be used for standard routine quality assessments of *Aedes* mosquitoes required for an SIT and other male release-based programs.

## 1 Introduction

The mosquito is one of the world’s deadliest animals. The Asian tiger mosquito *Aedes albopictus* (Skuse) , together with *Aedes aegypti* (Linnaeus) are highly invasive (Benedict et al., 2007) and medically important mosquito species that transmit several arboviruses and associated diseases including dengue, chikungunya, yellow fever, and Zika (Bhatt et al., 2013; Levy Blitchtein and Del Valle Mendoza, 2016).

There are a limited number of effective vaccines or drugs to protect against these diseases; thus, current disease control strategies rely on Aedes vector control, where the focus is usually on source reduction and the use of insecticides. However, the variability in the type of *Aedes* larval breeding sites and the presence of a myriad of cryptic breeding habitats coupled with the spread of insecticide resistance (Moyes et al., 2017) has created an even greater global need for alternative tools to control these disease vectors. Researchers have advocated for genetic control strategies, including the sterile insect technique (SIT) as an environment-friendly, insecticide-free technique. The SIT is based on inundated and repeated releases of sterile insects to induce sterility in the wild population to suppress the target pest species (Knipling et al., 1968). Great progress has been achieved over two decades in the development of the SIT package for mosquitoes. The Insect Pest Control Laboratory (IPCL) of the joint Food and Agriculture Organization/International Atomic Energy Agency (FAO/IAEA) Centre of Nuclear Techniques in Food and Agriculture has been leading the development of methods and guidelines, including mass-rearing equipment, irradiation, packing, transport, quality control and release for *Anopheles* and *Aedes* mosquitoes for deployment in field projects in member states reviewed in Vreysen et al. (2021).

Historical data have shown the achievements of SIT against several pests and vectors (Dyck et al., 2021). However, Benedict and Robinson (2003) have shown that several trials conducted in the 1970s had limited success due to the poor competitiveness of released males. Therefore, the quality of released males is critical to the success of SIT within an area-wide integrated pest management program (Parker et al., 2021). Several factors, including colonization, mass-rearing, irradiation, handling, and releases, may affect the quality of males in terms of their capacity to disperse, survive, locate females, and compete for mates (Bouyer and Vreysen, 2020; Parker et al., 2021). Important factors that impact the quality of male mosquitoes such as flight performance can only be assessed by time-consuming methods such as male mating competitiveness assay in the laboratory and mark–release–recapture (MRR) in the field. In addition, MRR experiments provide low recapture rates (Bellini et al., 2010; Bouyer et al., 2020; Iyaloo et al., 2020) and are tedious and costly, while conventional mating competitiveness assay is not only laborious but time-consuming. To circumvent these difficulties, a quick, easy-to-perform, and reliable quality control (QC) method to use before adult mosquitoes are released would be highly beneficial.

Flight is one of the most important traits of mosquito life history. Flight ability (capacity to escape or to fly) has been shown as a critical QC factor in fruit fly factories. Understanding this factor has helped to reduce production costs by improving rearing techniques in Mediterranean fruit fly mass-rearing facilities and has improved the level of competitiveness in released males (Shelly et al., 2010). Rowley et al. (1968) designed a flight mill, and other authors developed a video tracking system (Boyer et al., 2013; Lebon et al., 2018) to measure insect flight speed and the distance flown. Recent studies have developed mosquito QC tools based on a mosquito’s ability to escape from a flight test device (FTD). While some of the devices developed were based on young adults that had directly emerged from pupae (Balestrino et al., 2017), others used two-to-three-day-old adult mosquitoes (Culbert et al., 2018; Dor et al., 2020). Modified gray-colored polyvinyl chloride (PVC) flight tubes with different diameters and heights were tested to ascertain whether mosquito age or the height of the FTD would impact male *Ae. aegypti* escape rates (Dor et al., 2020). These modifications resulted in higher escape rates in all cases after 24 h. The FAO/IAEA reference QC FTD has been successfully tested in terms of assessing the flight performance of irradiated males and males under cold stress conditions after 2 hours (Culbert et al., 2018). Although preliminary tests were performed to design the FTD prototype (Culbert et al., 2018), further tests are needed in order to fine-tune the FTD for improved sensitivity and accuracy. These further tests include assessing the role of fan speed (airflow), the test duration, the density of males, the color of the flight tubes, the lure [Biogents (BG) pellets], fan or the presence of fan, lure, the mosquito strain origin, and the number of flight tubes within the device. In addition, the number of mosquito SIT programs is growing (Bouyer et al., 2020; WHO and IAEA, 2020), and facilities are in need of a standardized and efficient QC test. Several research groups have tested the prototype FTD and found inconsistent results and difficulties running the test. Therefore, this study aims to modify the original FTD to measure its efficiency while improving handling operation and test sensitivity and repeatability, while reducing its overall production cost. Factors in the test design that could affect the flight test’s applicability were investigated in order to standardize the device and user protocol.

## 2 Materials and Methods

### 2.1 Mosquito Strains, Rearing, Irradiation, and Cold Stress Conditions

Standard laboratory reference strains of *Ae. aegypti* and *Ae. albopictus* (FAO/IAEA, 2017, 2020) were used for all experiments. The *Aedes* strains were maintained following the “Guidelines for Routine Colony Maintenance of *Aedes* Mosquitoes” (FAO/IAEA, 2017) (*Experiments 2.3 to 2.9*). *Aedes aegypti* and *Ae. albopictus* strains originating from Brazil (Juazeiro) and Italy (Rimini) were transferred to the IPCL from the insectary of Biofabrica Moscamed, Juazeiro, Brazil, and from the Centro Agricoltura Ambiente, Bologna, Italy, in 2012 and 2018, respectively. These two institutions are IAEA collaborating centers for the development of mosquito SIT. These strains were used to assess factors that might affect mosquito flight ability (*Experiments 2.3–2.9*). Two recently colonized *Ae. albopictus* strains originating from Spain (Valencia, TRAGSA) and China (Guangzhou, Wolbaki) were used to assess flight ability at the IPCL (*Experiment 2.9*). In addition, a *w*AlbB strain of *Wolbachia*-infected *Ae. aegypti* (hereafter referred to as the “Singapore strain”) was independently maintained (Cheong Huat Tan, personal communication) at the Environmental Health Institute of the National Environment Agency, Singapore, to replicate two sets of standardization experiments (*Experiments 2.6–2.7*).

The rearing period had controlled conditions as follows: temperature of 28 ± 2°C, 80 ± 10% relative humidity (RH), and lighting of 14:10 h light: dark, including 1 h of dawn lighting and 1 h of dusk lighting for larval stages. Adults were separately maintained under 26 ± 2°C, 60 ± 10% RH, and 14:10 h light: dark, including 1 h dawn and 1 h dusk.

To perform the experiments, mosquitoes were reared following modified mass-rearing procedures developed at the IPCL (Mamai et al., 2019; Maïga et al., 2019; FAO/IAEA, 2020). Larval rearing started on Thursdays (day zero) when eggs were hatched and transferred to mass-rearing trays previously filled with 5 L of osmosis water on Fridays (day one). No larval feeding was performed during weekends and pupae were collected on day six after egg hatching. A 4% IAEA larval diet was provided daily: a 300 ml (0.66 mg/larva) meal on day one, a 300 ml (0.66 mg/larva) meal on day four, and a 200 ml (0.44 mg/larva) meal on day five. Larvae and pupae were separated on day six. Four liters of larval water were reused with an additional 300 ml for day six of larval rearing. Pupae were collected daily and sex-separated using mechanical and semiautomatic pupal sex sorters (John W. Hock Co., Gainesville, FL; Wolbaki, China).

For each experiment, pupae were aliquoted into 100 ml plastic cups (Medi-Inn, United Kingdom), each holding 110 male pupae and placed in cages (15 × 15 × 15 cm, BugDorm, BD4M1515, Taiwan) for emergence. About 100 (accounting for emergence and mortality rates) adults were maintained with access to a 10% sucrose solution until the day of the experiments.

To assess the sensitivity of the new FTD (version 2.0) (*Experiment 2.9*), three-to-four–day-old adult *Aedes* mosquitoes were exposed to cold stress conditions (4°C for 2 hours), which is known to significantly impact the flight ability of both *Ae. aegypti* and *Ae. albopictus* (Culbert et al., 2018). Males were allowed to recover for 2 h in the presence of a 10% sucrose solution prior to the test.

To assess the sensitivity of the new FTD (version 2.0) (*Experiment 2.9*), three-to-four–day-old adults were exposed to high-dose irradiation (100 Gy using an X-ray blood irradiator (Raycell MK2) (Gómez-Simuta et al., 2021) for *Ae. albopictus* and 150 Gy using the GammaCell 220 (Nordion Ltd., Kanata, Ontario, Canada) for *Ae. aegypti*), which is known to reduce the quality of mosquitoes (Culbert et al., 2018). Males were knocked down and held in a cold room at 4°C in compacted batches of 100/cm^3^ to simulate mass-transport conditions prior to irradiation. Males were allowed to recover for 2 h with a 10% sucrose solution prior to the flight ability test. Untreated (nonirradiated) male mosquitoes were kept in laboratory conditions with a 10% sucrose solution during both the cold stress and the irradiation treatments.

The test conditions for all experiments were 26 ± 2°C and 60 ± 10% RH, under a laboratory daylight regime (500–1000 lux), using untreated male mosquitoes (unless stated otherwise).

### 2.2 Modification of the Original Flight Test Device and Operation

**FIGURE 1:**
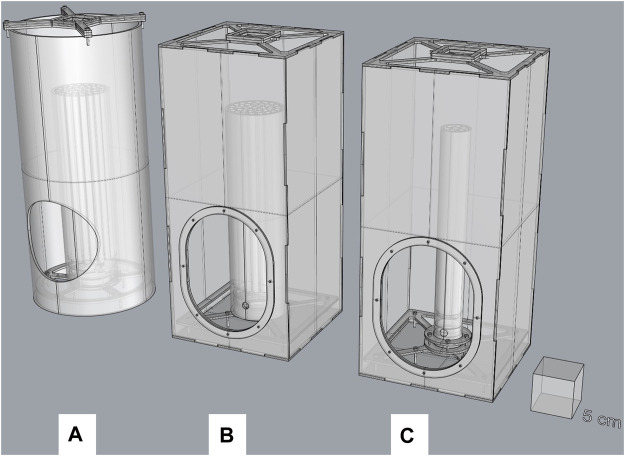
3D designs of the 40-tube original (version 1.0) **(A)** vs. the new FTDs: 40-tube FTD (version 1.1) in the middle **(B)**; eight-tube FTD (version 2.0) on the right **(C)**.

The original FTD (Culbert et al., 2018) (version 1.0) was modified in several ways to measure its efficiency while improving handling processes. The original FTD consists of 40 transparent acrylic plastic (polymethyl methacrylate, PMMA) tubes (hereafter referred to as “individual internal tubes”) that were placed together within a larger PMMA tube (hereafter referred to as the “inner tube”). Gaps between individual internal tubes were filled with transparent silicone (OBI, Austria). When male mosquitoes escape from the flight device, they can be recollected in a larger cylindrical PMMA tube (hereafter referred to as the “containment box”), which is closed at the top end with mesh (Figure 1A and Supplementary Material S1). A fan (DC axial fan: 40 mm, Vapo: 12 V, airflow: 0.218 m³/min, acoustic noise: 20.6 dB, and rated speed: 6,000 rpm, Multicomp, United Kingdom) and a BG-Lure (Biogents, Regensburg, Germany) pellets holder made of PMMA is placed on top of the end mesh.

**FIGURE 2:**
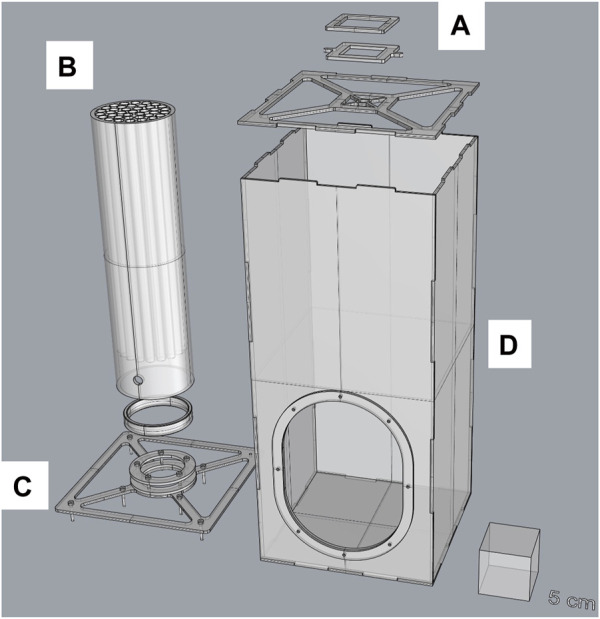
Split design of the new 40-tube flight test device (version 1.1). The device comprises a top cover **(A)** that can hold a fan and BG-Lure pellets, a flight test system (inner tube) **(B)** with 40 individual internal tubes, an inner tube holder (base) **(C)**, and the main container (containment box) **(D)**. More details are provided in the technical drawings (Supplementary Materials S2–S12).

To operate, the inner tube was inserted and removed from the bottom of the containment box, which was secured with a mesh sleeve. Mosquitoes were first loaded into the FTD before the device was introduced through the bottom in the larger cylindrical tube because the side opening was too small to allow side introduction. In addition, after the test was completed, the device was taken to a cold room to knock down the mosquitoes prior to counting. These handling processes were found to be tedious for nonexperienced operators and may have increased the risk of damaging the FTD. Therefore, we designed a square containment box to replace the larger cylindrical tube. The containment part of the device is easily opened from the top, and the inner tube can be inserted or removed more easily through a larger opening on the front side of the containment box [Figure 1B, Figure 2 (version 1.1)].

In addition, another version of the FTD was developed. The new FTD (version 2.0) (Figure 1C) is made of eight individual internal tubes fitted in an inner tube of 4 cm in diameter. The tube holder (base) was resized to fit the inner tube better. The containment box and the tube length are all similar to version 1.1.

For all experiments, the following steps were followed to perform the flight test (Supplementary Materials S13, S14). Before any test, one to two pellets (10–30 mg) of BG-Lure (Biogents, Regensburg, Germany) were placed on top of the FTD. A fan was placed directly on the small BG decoy cap and switched on at its standard speed (3000 rpm unless stated otherwise). About 100 adult male mosquitoes (unless stated otherwise) were aspirated using a mouth aspirator and were blown into the FTD *via* a small 1 cm hole at the bottom of the device. Males were then confined within a small space (height × diameter: 1 × 7 cm) and flew upwards through one of the individual internal tubes and out into the large containment box. After 2 hours, the top of the inner tube was covered using a Petri dish (9 cm in diameter) to avoid further escapes, the fan was stopped, and the experiment was deemed as complete. The inner tube was then removed through the large opening of the containment box by tilting it slowly. When tilted halfway, the Petri dish covering the top of the tube was held to avoid losing it. To remove the nonescaped males from the inner tube, mosquitoes were blown through the bottom into a cage (15 × 15 × 15 cm, BugDorm, BD4M1515, Taiwan). The escaped males were removed using a mouth (or mechanical) aspirator from the containment box and transferred to another cage (15 × 15 × 15 cm, BugDorm, BD4M1515, Taiwan). All cages were taken into a freezer (−20°C) for 20–30 min and numbers were counted for each treatment.

### 2.3 Effects of Fan Speed or Airflow on Escape Rate

To assess whether the fan speed could affect a mosquito’s capacity to escape, two fan speeds were tested. The FTD fan (Multicamp, United Kingdom) was set to either its highest speed (high, 6000 rpm) or at its standard speed (normal, 3000 rpm). Three FTDs (version 1.1) were tested with two-to-three–day-old male *Ae. aegypti* (Brazil strain) mosquitoes and four FTDs with four-to-five–day-old male *Ae. albopictus* (Rimini, strain) mosquitoes for each speed, respectively.

### 2.4 Effects of Test Duration on Escape Rate

It was initially observed that as males were being loaded into the FTD, a large number of mosquitoes escaped immediately. Therefore, to ascertain a suitable length of time to perform the QC flight test, a range of times were tested—including 30, 60, 90, and 120 min. The test was repeated four times (with two, two, two, and three pseudoreplicates per repetition, respectively) with two-to-three–day-old male *Ae. aegypti* (Brazil strain) and twice (two pseudoreplicates each) with two-to-three–day-old male *Ae. albopictus* (Rimini, strain) for each duration.

### 2.5 Effects of Male Density on Escape Rate

Densities of 25, 50, 75, and 100 males were tested to see whether the number of males loaded into an FTD would affect the output of the escape rate. The test was performed with four FTDs for each density with two-to-three–day-old male *Ae. aegypti* (Brazil strain) mosquitoes. For *Ae. albopictus* (Rimini strain) mosquitoes, the density effect was assessed twice (four and two pseudoreplicates) with two-to-three–day-old males for each density.

### 2.6 Effects of Internal Tube Color and Addition of Lure and Fan on Escape Rate of *Aedes aegypti* and *Aedes albopictus*

To assess whether the color of the FTD could affect escape rates, two types of inner tubes (where the tube ends with transparent and pink silicon) were used (Supplementary Material S15). For each tube type (transparent and pink), the presence/absence of fan and lure was tested for *Ae. aegypti* (Brazil strain) and *Ae. albopictus* (Italy strain): one with the addition of BG-Lure only (pink/transparent + lure), one with fan only (pink/transparent + fan), one with fan and lure together (pink/transparent + fan + lure), and one FTD without a fan or lure (pink/transparent). The test was performed twice (four and two pseudoreplicates/treatment) with two-to-three–day-old male *Ae. aegypti* and with two-to-three–day-old male *Ae. albopictus* mosquitoes, respectively.

The same experiment was carried out with five-to-six–day-old male *Aedes aegypti* in Singapore (Singapore strain) using only the transparent FTDs (version 1.1). The escape rates from two FTDs with fan and lure (transparent + fan + lure) were compared to three FTDs of each of the following: with lure only (transparent + lure), with fan only (transparent + fan), and without fan and lure (transparent).

For the FTD to be tested without a fan (absence of a working fan), a fan was still placed on top of the device but without turning it on.

### 2.7 Effects of Male Adult Age on Flight Ability

The effect of age on male flight ability was assessed for different age groups ranging from less than 1 day up to 8 days old for *Ae. aegypti* (three replicates/age group) and from less than 1 day up to 10 days for *Ae. albopictus.* In addition, two age groups (12–13 and 16–17 days) were assessed for the latter*.* Four replicates were performed for each *Ae. albopictus* age group.

The same experiment was repeated at the Environmental Health Institute, National Environment Agency, Singapore. Male *Ae. aegypti* (Singapore strain), aged between two to six days, were tested. Four age groups, including two to three, three to four, four to five, and five to SIX days, were used to assess and compare mosquitoes’ ability to fly using the 40-tube transparent FTD (version 1.1). The flight test was performed in total 21, nine, nine, and 27 times for the two-to-three-, three-to-four–, four-to-five–, and five-to-six–day-old mosquitoes, respectively.

### 2.8 Assessing the Flight Ability of *Aedes albopictus* Strains From Different Origins

To assess whether different strains of *Ae. albopictus* exhibit different flight ability scores using the 40-tube FTD (version 1.1), three strains from different origins, Italy (Rimini), Spain (Valencia), and China (Guangzhou), were evaluated. The flight test was repeated twice with three pseudoreplicates per repetition per strain with three-to-four–day-old males. Male mosquitoes were randomly selected from each of the six pseudoreplicates per strain (Rimini: 82; Valencia: 84; and Guangzhou: 79 mosquitoes in total), and their right wings were dissected and measured as a proxy for strain adult size (Nasci, 1990).

### 2.9 Effects of Fewer Internal Flight Tubes on Escape Rate

To assess whether a reduction of the number of individual internal tubes within the flight test inner tube could affect the performance of the FTD (version 1.1), a FTD was made containing eight individual internal tubes fitted in an inner tube with a diameter of 4 cm (version 2.0). The eight-tube FTD was also tested with both mosquito species. In addition, the effects of cold stress (4°C chilling for 2 hours) and high-dose irradiation (100 and 150 Gy for *Ae. albopictus* and *Ae. aegypti*, respectively) (see Section 2.1 *.*) on the sensitivity of the new FTD (version 2.0) were evaluated.

#### 2.9.1 Data Analysis

The escape rate in each experiment was analyzed using a generalized binomial linear mixed-effects model fit by maximum likelihood (Laplace approximation) with a logit link, with the escape rate (proportion of flyers) defined as the dependent variable [whereby escaped (success or flyers) and nonescaped (failure or nonflyers) were weighted with the “cbind ()” function] and replicates as a random effect, considering inferences needed to be done independently of their levels in our specific experimental design (Chaves, 2010).

The fan speed (two levels: normal and high), the test duration (four levels: 30, 60, 90, and 120 min), the adult density per flight test (four levels: 25, 50, 75, and 100 male mosquitoes) were successfully considered as fixed effects in separate models.

To determine the effects of tube color (pink/transparent) and the presence/absence of lure and fan on the escape rates, the tube color, the lure, and fan (coded as “1” for presence and “0” for absence) were considered as fixed effects for each mosquito species/strain. In addition, to assess the effect of a combination of factors defined as flight test treatment (four levels: fan only, lure only, no fan and no lure, and fan and lure for each tube color) on escape rate, treatment was considered as a fixed effect.

Mosquito age (seven and 12 levels for *Ae. aegypti* and *Ae. albopictus*, respectively) was considered as a fixed effect to analyze the effects of age on male flight ability. In addition, four levels (two to three, three to four, four to five, and five to six days) were considered to analyze the effects of age in *Ae. aegypti* (Singapore strain).

Male *Ae. albopictus* mosquito strain (three levels: Rimini, Valencia, and Guangzhou) was considered as a fixed effect to compare flight ability based on origin.

The number of individual internal flight tubes (two levels: 40-tube FTD and eight-tube FTD) and treatments (three levels: chilled, irradiated, and control) were considered as fixed effects to analyze the effect of FTD type and cold and high-dose irradiation stress conditions on adult flight ability.

For validation, the full models were checked for overdispersion (using Bolker’s function) (Bolker, 2018) and for normality and homogeneity of variances on the residuals (Kéry and Hatfield, 2003). When overdispersion in model fit (glmer function) was detected, an individual level random variable was created and added to the model (Harrison, 2015). However, when overdispersion was detected in model fit (glm function), an analysis was performed using quasibinomial errors. The stepwise removal of terms followed by likelihood ratio tests (LRTs) or based on the lowest value of Akaike's Information Criterion (AICc) was used for model simplification. The minimal adequate model retained only factors that significantly reduced explanatory power (*p* < 0.05) when removed (Crawley, 2012). Differences between the levels of significant fixed factors were analyzed using post hoc Tukey’s tests (glht function in package multcomp) (Bretz et al., 2016). The significant interactions were analyzed using the emmeans function (in package emmeans) (Lenth, 2020). All statistical analyses were performed using R version 4.0.3 (https://cran.r-project.org) using RStudio (RStudio, Inc. Boston, MA, United States, 2016). All significant differences are based on *p* < 0.05.

## 3 Results

### 3.1 Effects of Fan Speed or Airflow on Escape Rate

The fan speed (“normal” 3000 rpm vs. “high” 6000 rpm) had no effect on mosquitoes’ escape rate through the FTD in both *Ae. aegypti* [high: 0.76 (0.71–0.81, 95%CI), normal: 0.70 (0.65–0.75, 95%CI), χ^2^ = 1.17, df = 1, *p* = 0.28] and *Ae. albopictus* [high: 0.90 (0.87–0.93, 95%CI), normal: 0.91 (0.88–0.94, 95%CI), χ^2^ = 0.2, df = 1, *p* = 0.65].

### 3.2 Effects of Test Duration on Flight Ability

**FIGURE 3:**
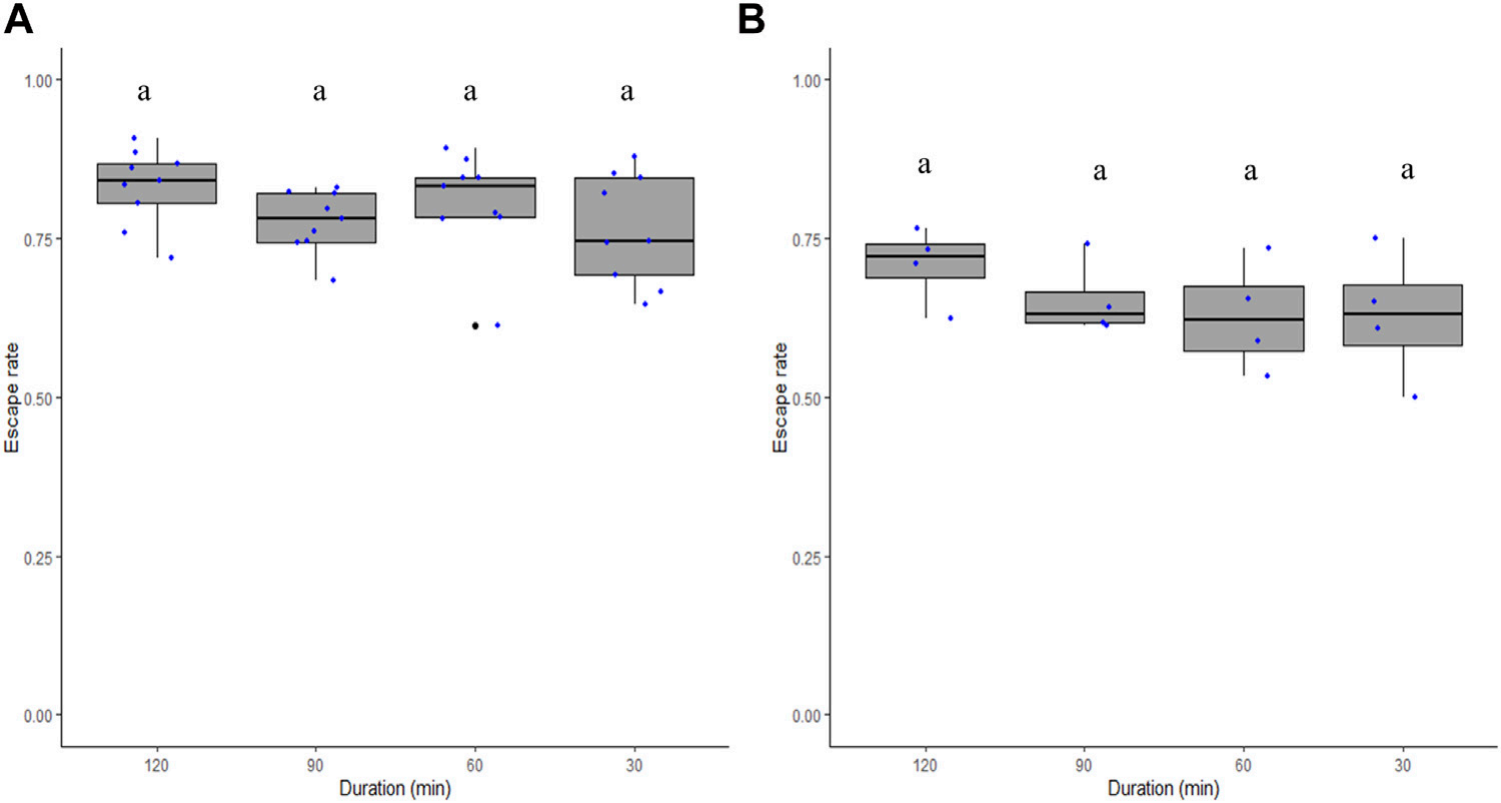
Male *Aedes aegypti* **(A)** and *Aedes albopictus* **(B)** escape rates in response to test duration. Four time periods of 30, 60, 90, and 120 min were tested. Different letters denote significant differences between time periods.

The duration of the flight ability test did not significantly differ between 30, 60, 90, and 120 min in both *Ae. aegypti* (χ^2^ = 5.35, df = 3, *p* = 0.14; Figure 3A) and *Ae. albopictus* (χ^2^ = 3.57, df = 3, *p* = 0.31; Figure 3B).

### 3.3 Effects of Male Density on Escape Rate

**FIGURE 4:**
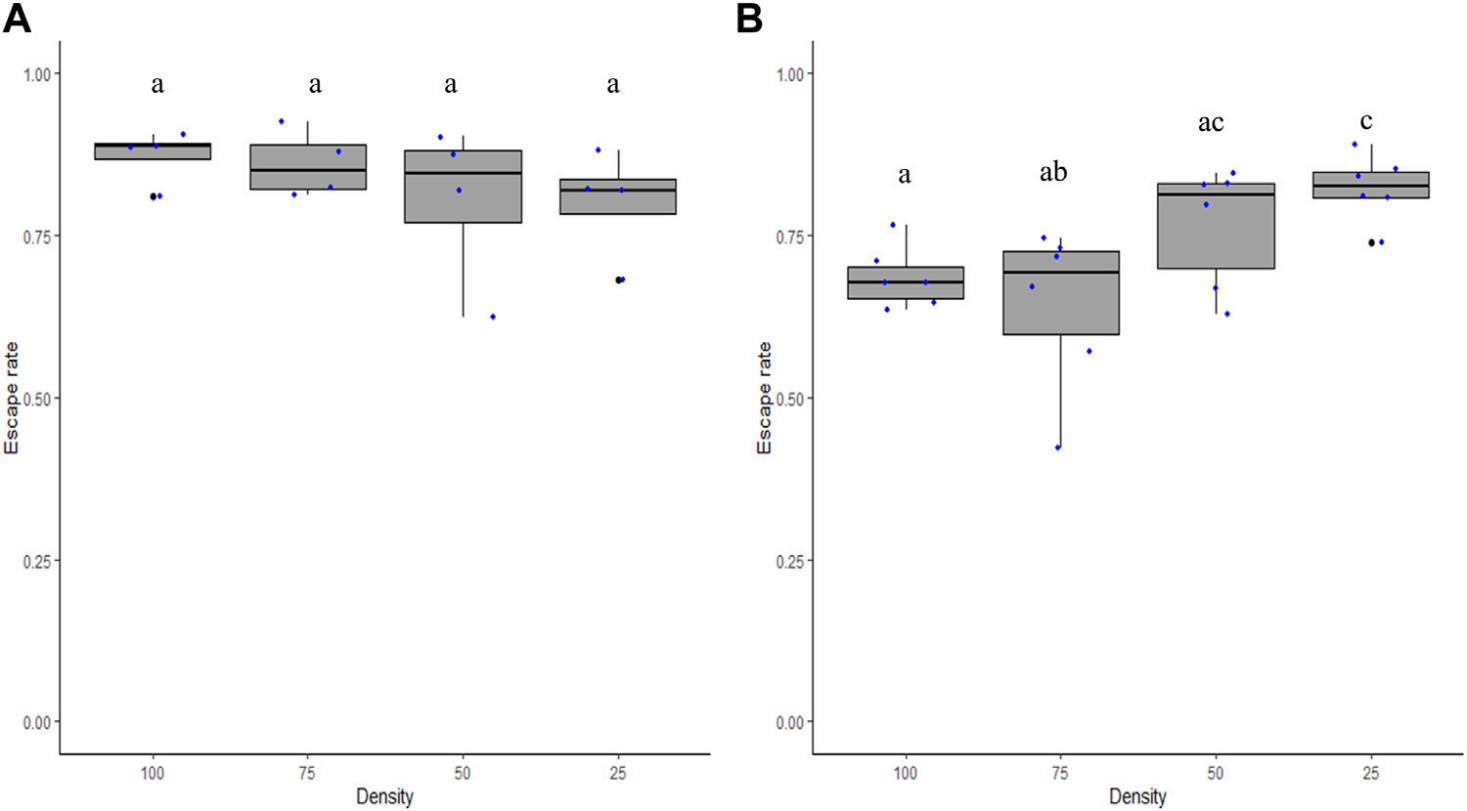
Escape rates according to male density for *Aedes aegypti* **(A)** and *Aedes albopictus* **(B)**. Numbers (25, 50, 75, and 100) stand for the adult male density loaded in each flight test device. Different letters denote significant differences between densities.

The densities of male mosquitoes within the FTD between 25 and 100 did not impact escape rates in *Ae. aegypti* (χ^2^ = 6.3, df = 3, *p* = 0.09; Figure 4A) but had a significant impact in *Ae. albopictus* (χ^2^ = 26.2, df = 3, *p* = 0.0001; Figure 4B). The Tukey test shows that a density of 25 male *Ae. albopictus* led to a greater mean escape rate than that of the densities 75 and 100 (*p* < 0.05).

### 3.4 Effects of Internal Tube Color and Addition of Lure and Fan on Escape Rate of *Aedes aegypti* and *Aedes albopictus*

Greater number of male *Ae. aegypti,* the Brazilian strain, and *Ae. albopictus*, the Italian strain, escaped from the transparent tube more than those that escaped the pink-colored internal tube (*p* < 0.001, Table 1). There was a greater impact of the fan on the escape rate of *Ae. aegypti*, Brazilian strain (*p* = 0.03, Table 1), and *Ae. aegypti*, Singaporean strain (*p* < 0.001, Table 1). The presence of a lure did not enhance the escape rate of both *Ae. aegypti* strains (*p* > 0.05, Table 1), but there was a significant interaction between fan and lure on the escape rate of *Ae. albopictus*, Italian strain (*p* = 0.01, Table 1).

**TABLE 1:** Fixed effects of the internal tube color and addition of lure and fan (or none) on the escape rates of *Aedes aegypti* (Brazilian strain), *Aedes albopictus* (Italy, Rimini strain), and *Aedes aegypti* (Singaporean strain) males.

Species/strain	Factors	Estimate	Std. error	z value	Pr(>|z|)
*Aedes aegypti*, Brazil	(Intercept)	0.5765	0.155	3.719	0.0002***
	Transparent	0.342	0.1169	2.925	0.00344**
	Fan1	0.2673	0.1244	2.149	0.03162*
	Lure1	0.2085	0.1283	1.624	0.10427
	(Intercept)	−0.4517	0.1612	−2.803	0.00507 **
	Transparent	1.1402	0.123	9.271	<2e-16 ***
*Aedes albopictus*, Italy	Fan1	−0.1326	0.1777	−0.746	0.45575
	Lure1	−0.5045	0.2922	−1.726	0.08429
	Fan1 × lure1	0.8285	0.3279	2.527	0.01151 *
	(Intercept)	0.2998	0.11897	2.52	0.01174 *
*Aedes aegypti*, Singapore	Fan1	0.52074	0.17265	3.016	0.00256 **
	Fure1	0.01417	0.16834	0.084	0.93292

Signif. codes: 0 “***” 0.001 “**” 0.01 “*” 0.05 “.” 0.1 “ “ 1.

The number “1” following fan and lure stands for their presence as compared to the absence of fan and lure (coded as “0”). The effect of the internal tube color “transparent” was compared to the pink-colored tube.

**FIGURE 5:**
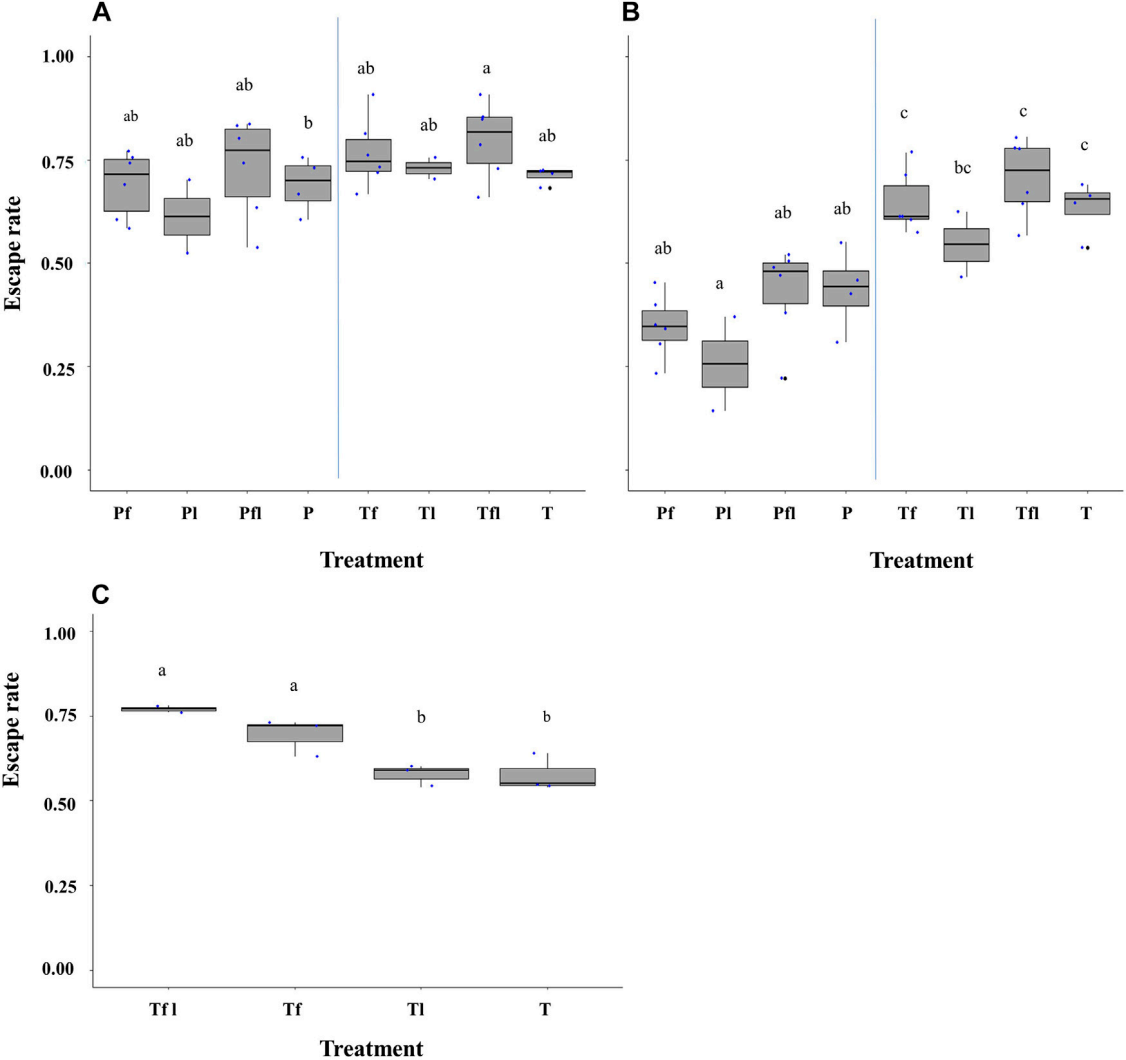
Comparison of escape rates according to the flight tube color and treatment (pink, transparent tubes, with/without lure, with/without fan, and with/without lure and fan) in *Aedes aegypti* (Brazilian strain) **(A)** and *Aedes albopictus* **(B)** and *Aedes aegypti* (Singaporean strain) **(C)**. Pink (P) and transparent (T) stand for flight test device (FTD) tube color (i.e., tube ends with pink silicon or transparent silicon). fl = fan and lure; f = fan only; l = lure only; P/T = pink or transparent without fan and lure. Black bars indicate the median; the upper and lower limits of each box indicate the interquartile range. Each dot represents a value of the observed escape rate per replicate. Different letters denote significant differences between treatments.

When considering the treatment of the FTD (i.e., with/without lure, with/without fan, and with/without lure and fan), a significant impact was found on the escape rate of *Ae. aegypti,* Brazilian strain (χ^2^ = 14.8, df = 7, *p* = 0.03; Figure 5A), *Ae. albopictus*, Italian strain (χ^2^ = 48.5, df = 7, *p* < 0.001, Figure 5B) and *Ae. aegypti,* Singaporean strain (χ^2^ = 29.04, df = 3, *p* < 0.001; Figure 5C). Furthermore, a higher number of mosquitoes escaped from the reference FTD treatment where a transparent tube, a working fan, and lure were simultaneously used (Figure 5). There was a similar number of escapes from the pink-colored FTD in *Ae. aegypti*, Brazilian strain (Figure 5A), and *Ae. albopictus*, Italian strain (Figure 5B) regardless of the treatment. On the other hand, the difference in the escape rates between the pink-colored tube and the transparent tube tends to be greater in *Ae. albopictus* (Figure 5B) than that in *Ae. aegypti* (Figure 5A). Although more escapes were recorded from the FTD with fan and lure, they were not significantly different from those from the FTD with a working fan (Figure 5). The FTD baited with a lure or without lure and fan displayed lower escape rates than those of the reference FTD and the FTD with a working fan (Figure 5C).

### 3.5 Effects of Male Adult Age on Flight Ability

**FIGURE 6:**
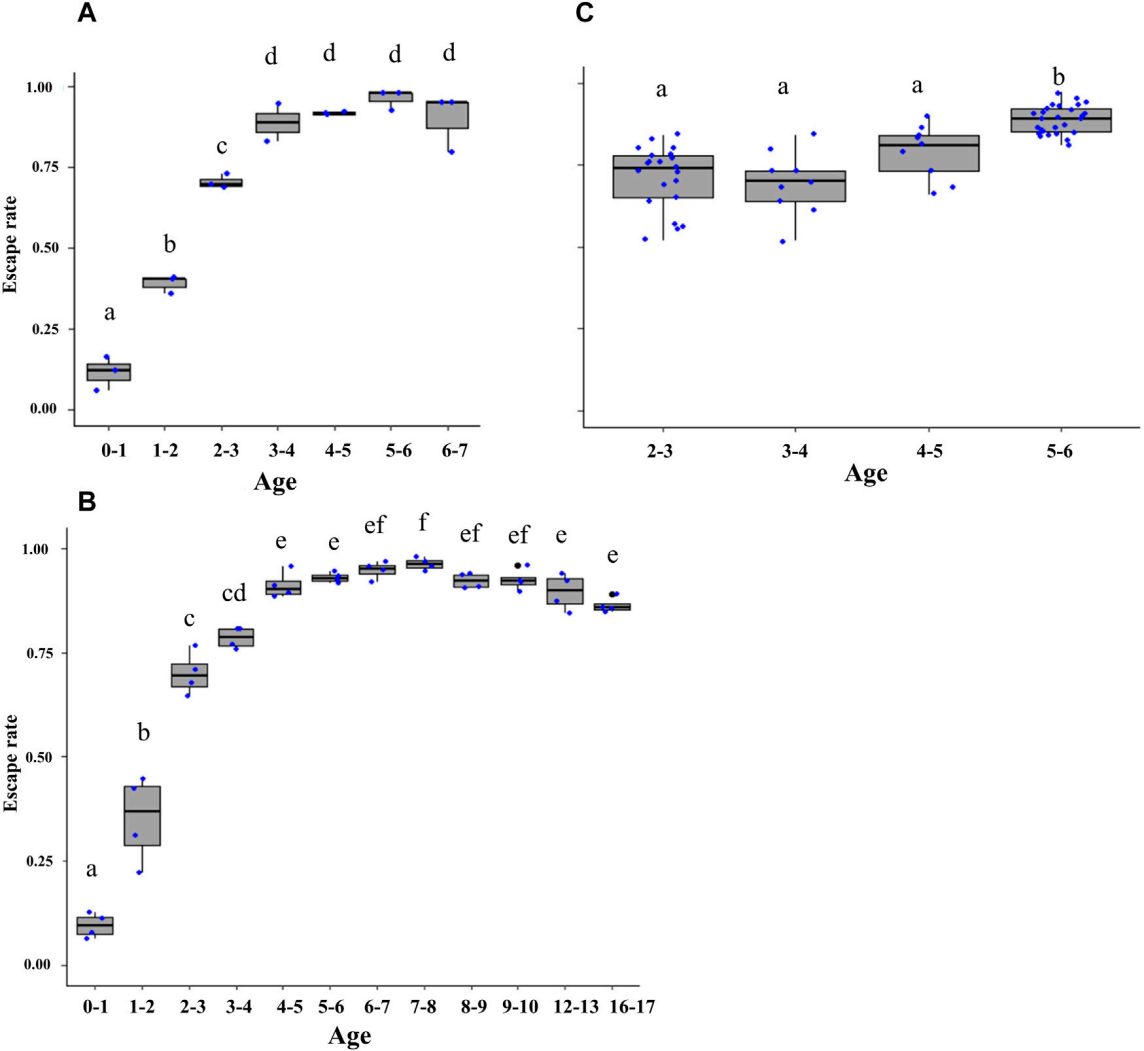
Escape rates across adult male *Aedes aegypti* (Brazilian strain) **(A)**, *Aedes aegypti* (Singaporean strain) **(B)**, and *Aedes albopictus* **(C)** age groups. Age ranged from below one to eight days old **(A)**; from below 1 day to 17 days **(B)**; from two-to-six–day-old mosquitoes **(C)**. Black bars indicate the median; the upper and lower limits of each box indicate the interquartile range. Each dot represents a value of the observed escape rate per replicate. Different letters denote significant differences between age groups.

Age had a significant effect on male flight ability in both *Ae. aegypti* (Brazil strain) (χ^2^ = 62.7, df = 6, *p* < 0.001; Figure 6A) and *Ae. albopictus* (χ^2^ = 1684.4, df = 11, *p* < 0.001; Figure 6B). The pairwise comparison of means showed that two-to-three–day-old male *Ae. aegypti* (reference age in Culbert et al., 2018) had higher flight ability than those younger than 2 days (*p* < 0.001) but lower flight ability than males older than 3 days (*p* < 0.001). Similarly, two-to-three–day-old and three-to-four–day-old male *Ae. albopictus* had significantly higher flight ability than those younger than 2 days (*p* < 0.001) but lower than males older than 4 days (*p* < 0.001). Flight ability of male *Ae. aegypti* and *Ae. albopictus* declined after the age of five to six and seven to eight days, respectively, leading to a significant decrease in 16-17–days-old male *Ae. albopictus* of (*p* < 0.001) as compared to seven-to-eight–day-old males. Similarly, age had a stronger impact on flight ability in male *Ae. aegypti* (Singapore strain) (χ^2^ = 62.5, df = 3, *p* < 0.001; Figure 6C).

### 3.6 Assessing the Flight Ability of *Aedes albopictus* Strains From Different Origins

**FIGURE 7:**
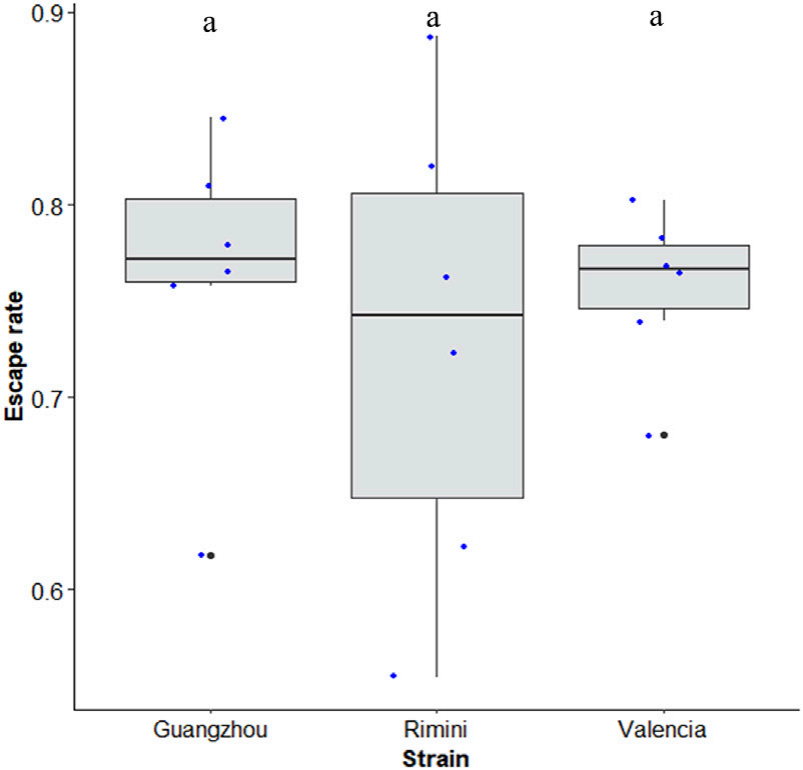
Escape rate of *Aedes albopictus* strains from different origins (Rimini, Italy; Valencia, Spain; and Guangzhou, China). Black bars indicate the median; the upper and lower limits of each box indicate the interquartile range. Each dot represents a value of the observed escape rate per replicate. Different letters denote significant differences between strains.

Similar escape rates of about 74 ± 20 (±95% CI) were observed when different strains of *Aedes albopictus* from different origins (Rimini in Italy; Valencia in Spain; Guangzhou in China) were tested using the 40-tube FTD (χ^2^ = 0.68, df = 2, *p* = 0.70) (Figure 7).

Wing length of male mosquitoes varied significantly between *Ae. albopictus* strains from different origins (χ^2^ = 57.03, df = 2, *p* < 0.001, Figure 7). *Aedes albopictus* (Guangzhou strain) exhibited a higher body size as compared to Valencia and Rimini strains (*p* < 0.01), whereas the other two strains have similar sizes (*p* = 0.77, Supplementary Figure S1)**.**

### 3.7 Effects of Fewer Internal Flight Tubes on Escape Rate

**FIGURE 8:**
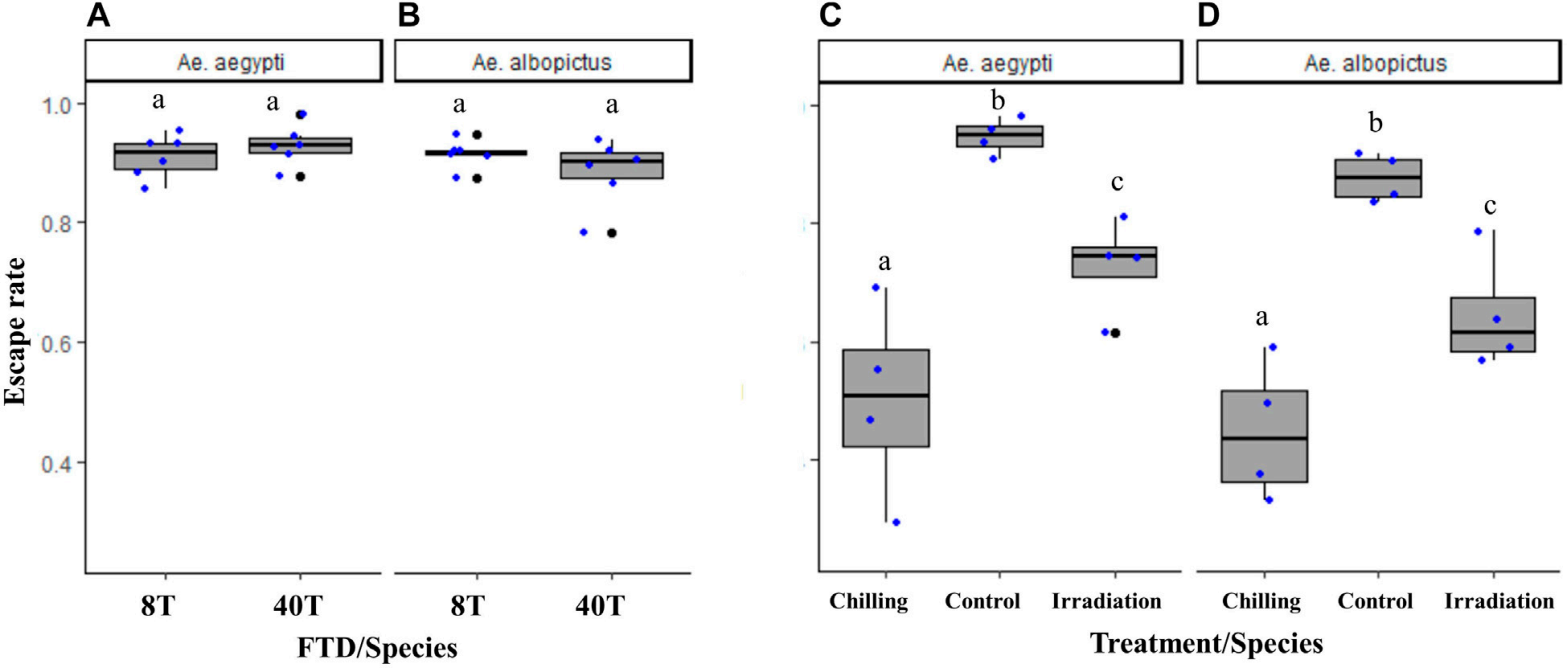
Escape rates of male *Aedes aegypti* **(A)** and *Aedes albopictus* **(B)** in eight-tube FTD (“8T”: version 2.0) versus 40-tube FTD (“40T”: version 1.1) and effects of cold stress (“chilled”) and high-dose irradiation (150 Gy, GammaCell and 100 Gy, X-ray, Raycell) on male *Aedes aegypti* **(C)** and *Aedes albopictus* **(D)** flight ability, respectively, using the eight-tube FTD. Black bars indicate the median; the upper and lower limits of each box indicate the interquartile range. Each dot represents a value of the observed escape rate per replicate. Different letters denote significant differences between FTDs and treatments.

When the new FTD with eight individual tubes (version 2.0) was compared to the 40-tube FTD (version 1.1), similar flight capacity was observed both in *Ae. aegypti* (χ^2^ = 1.26, df = 1, *p* = 0.26; Figure 8A) and *Ae. albopictus* (χ^2^ = 2.86, df = 1, *p* = 0.09; Figure 8B).

The eight-tube FTD (version 2.0) was as sensitive to stress conditions such as cold temperature and high irradiation doses in both *Aedes* mosquito species as the 40-tube FTD (version 1.1) (*Ae. aegypti*: χ^2^ = 23.44, df = 2, *p* < 0.001; *n* = 6, Figure 8C; *Ae. albopictus*: χ^2^ = 22.38, df = 2, *p* < 0.001, *n* = 6, Figure 8D).

## 4 Discussion

Our findings demonstrated that the flight tube treatment, including the use of BG-Lure, the use of transparent tubes, and the fan, for a period of 2 h provided consistent and reproducible results for a specified mosquito age group, thus allowing optimal use of the FTD. In addition, a cheaper FTD with only eight tubes was further assessed as a better value QC tool.

It is known that environmental factors, including visual or chemical cues detected by mosquitoes, can affect their behavior (Pitts et al., 2013). The FAO/IAEA reference QC FTD operates with a fan and BG-Lure. The question was whether it could run without either of these without impacting the observed escape rates of mosquitoes from the device. Various airflow rates through fan speeds set at 3000 or 6000 rpm had no effect on the mosquitoes’ ability to fly through the FTD, suggesting that this airflow falls within the range for standard operation of the FTD. The presence of a fan (i.e., fan only) showed an increase in mosquito escape rate in *Ae. aegypti* (Brazilian and Singaporean strains). This effect may simply be due to the airflow or the noise of the fan, which was measured at 20.6 dB. Fans are widely used to trap mosquitoes. Depending on the speed, fans produce a characteristic noise that could enhance trap attraction and efficiency (Swan et al., 2021). Dou et al. (2021) assessed the effects of incidental sound stimuli on the flight behavior of free-flying male vs. female *Ae. aegypti* and *Anopheles gambiae* mosquitoes and showed a relative increase in flight speed in response to the stimulus. Conversely, the FTDs baited with or without BG-Lure did not enhance the capacity of males to escape as compared to both the reference FTD and the FTD with a working fan but did reduce variability. This could be since all FTD treatments were run together in the same room and thus, we cannot rule out an additional effect (or interaction) of the lure. Similar results were observed when neither lure nor fan were provided. BG-Lures are blends of mosquito attractants consisting of lactic acid, ammonia, and caproic acid. These components are found on human skin (Kröckel et al., 2006) and are used to mimic human odor. To standardize the use of the FTD, BG-Lure was used to saturate mosquito receptors and reduce their sensitivity to the operators’ odor. Potential synergetic effects of the combination of lure and fan led to better escape rates in both *Ae. aegypti* and *Ae. albopictus* mosquito species in our study. Hapairai et al. (2013) showed a significant number of male *Ae. aegypti* collected in French Polynesia using odor-baited BG-Sentinel Traps, while several studies reported the use of BG-Lures in traps to catch male *Aedes* mosquitoes (Pombi et al., 2014; Amos et al., 2020; Visser et al., 2020), Staunton et al. (2021) found during a field study that BG-Lures did not significantly change catch rates and may have even repelled male *Ae. aegypti* and *Ae. albopictus*. A more recent study demonstrated that conflicting results for male *Ae. aegypti* mosquitoes’ attraction to humans could be linked to the experimental setting size and assay design for mosquito behavioral research (Amos et al., 2020).

Notably, greater escape rates were observed in *Ae. aegypti* than those in the *Ae. albopictus* mosquito species in our study, meaning that flight behavior may differ between species, highlighting the importance of control groups*.* Female *Ae. albopictus* were found to be weaker flyers than female *Ae. aegypti* (Briegel et al., 2001). Male *Ae. aegypti* may be more responsive to the device and to different stimuli, including BG-Lure and fan (or their absence), and may tolerate better-shadowed FTDs (Supplementary Material S16). These findings highlight the need to avoid any shadows or dark resting sites around the FTD as this may impact flight behavior and cause misleading results regarding mosquito quality based on the escape scores. Shade and vegetation were found to be important determinants of male *Ae. albopictus* catch rate success using BGS traps (Crepeau et al., 2013). In addition, when a black cloth of 8 cm in diameter was placed on top of the pink-colored FTD as an attractant against BG-Lure and fan, it did not improve the observed escape rate in both *Aedes* mosquito species (Supplementary Material S16). This also shows that although the color of the fan is black, it did not induce better escape rates.

A quick mosquito flight ability tool is preferable for routine use. Finding a minimum response time for the FTD is important. We found that although no difference in overall escapees for each time period (30, 60, 90, and 120 min) was observed, less variation between replicates was observed for 120 min. Indeed, once mosquitoes are blown into the FTD, they fly upwards through one of the individual internal tubes into the large containment box. However, a proportion of males could be seen resting on top of the inner tube. We cannot rule out that some mosquitoes may return into the individual tubes and so a minimum predetermined duration might be needed for consistent output. Dor et al. (2020) proposed time periods between one and 5 hours to be investigated using their device (80 × 2 cm). In any case, a 2 h time period would be acceptable for a rapid assessment of short-range flight activity as an indicator for their overall quality and performance once released into the field site. However, for this, determining a proxy of male mating capacity based on flight ability would be key. It has been shown that a flight ability score after 2 h could be predictive of survival and insemination rates with over 80% of the inertia (Culbert et al., 2018).

Recent studies on flight assays recommended using two-to-three–day-old adult male *Aedes* mosquitoes for QC (Culbert et al., 2018; Dor et al., 2020). However, it is known that age-related changes in mosquitoes’ flight muscles may occur (Johnson and Rowley, 1972) and this may impact flight ability (Rowley and Graham, 1968). Therefore, assessing the flight performance of males of different age groups could help guide the predicted peak age for flight potential for field releases. In our study, although different strains of *Ae. aegypti* from Brazil (maintained at the IPCL) and from Singapore were maintained in different conditions, including larval diet, they both exhibited a significant increase in ability to escape from the FTD with increasing age. This highlights the need for each facility to set up its own QC reference baseline escape rates. We have shown here that mosquito flight ability reference values should be based on a specific age range. Oliva et al. (2012) observed that male *Ae. albopictus* mosquitoes (La Reunion strain) sexual maturation was completed within 13–20 h postemergence and some males were able to inseminate females when 15 h old. However, one-day-old males were less competitive than five-day-old ones in laboratory conditions. Rowley and Graham (1968) indicated that the limiting factor in flight ability appears to be the extent of the glycogen reserves in young mosquitoes, but older mosquitoes are unable to utilize or mobilize glycogen in flight. Increased flight ability with age was also previously demonstrated in insects, including *Drosophila funebris* and *D. melanogaster* (Williams et al., 1943; Wigglesworth, 1949). The response to the FTD could be related to an interaction between age and attractants. This phenomenon was also observed when 10-to-15–day-old mosquitoes were more responsive to CO_2_ and human skin odor than younger (three-to-five days old) adults in both *Ae. albopictus* and *Culex quinquefasciatus* (Drago et al., 2021). Sexually immature *Ae. aegypti* males (under 24 h old) exhibit flying but not swarming behavior, according to Cabrera and Jaffe (2007). They also described that sexually mature males (above 24 h of age) initiate a small swarm and secrete an aggregation pheromone, which stimulates and attracts more conspecific males to the swarm. A flight mill assay did not show a difference in mean total flight capability (distance, duration, and velocity) in male *Culex pipiens pallens* (L.) in contrast to their female counterparts (Cui et al., 2013). We also observed that after the age of five to six and seven to eight days, flight ability declined with age in *Ae. aegypti and Ae. albopictus* mosquitoes, respectively. Aging is known as a factor that causes wing damage in house flies (Wehmann et al., 2022), which might impact flight ability.

The FTD has shown its capacity to measure the performance of male *Ae. albopictus* mosquitoes from different geographical origins but maintained in the same conditions. This shows that the device can be widely used in SIT-based control programs against these vectors. Recently, the FTD was used to assess the flight ability of long-term mass-reared *Ae. albopictus* and *Ae. aegypti* mosquito populations (Mamai et al., 2019; Somda et al., 2019; Li et al., 2021). Although there was a significant difference in adult body size between strains, no difference in escape rates was recorded, suggesting that a slight variation in size between strains reared in similar conditions might not limit the use of the FTD. Nevertheless, a recent study has shown that *An. arabiensis*, which are larger than *Aedes* mosquitoes, exhibited fewer escapes from the device. Consequently, a wider diameter of the individual internal tubes (from eight to 10 mm) was proposed for that species (Culbert et al., 2020).

Given the growing number of mosquito SIT programs (Bouyer et al., 2020), a number of IAEA member states are interested in a standardized easy to use and cheap QC method. One of the general challenges of the FTD is its cost. Indeed, the 40 individual tubes of the FTD comprise the majority of the costs of the device in itself. Reducing the number of tubes to eight led to a three- to fivefold reduction in price. A simpler short-flight range device could be designed and created with the aim of further reducing the overall cost while maintaining a 2 h time period for the routine QC assays. Alternative materials to lower the production cost of the flight device could also be investigated in developing countries. Modified gray-colored PVC single-flight tubes with different diameters and heights were tested for male *Ae. aegypti* escape rates after 24 h (Dor et al., 2020). A QC tool that measures mosquitoes’ capacity to fly should be sensitive enough to distinguish factors including high-dose irradiation (Parker et al., 2021) and cold stress (Culbert et al., 2019), as these factors are known to impact male mosquito quality. As such, the observed escape rates using the eight-tube FTD were consistent with similar studies performed with the 40-tube FTD (Culbert et al., 2018, 2020), and thus, we propose this cost-effective version as an alternative.

## 5 Conclusion

This study demonstrated that several factors may influence the measurement of flight performance using the FAO/IAEA reference FTD. The device should be used with a transparent middle flight tube to assess the quality of mass-reared *Ae. aegypti* and *Ae. albopictus* males. The test should be completed with the addition of BG-Lure and fan for a period of 2 hours for a specified mosquito age group. All experiments should be performed in similar conditions of daylight. As this factor may differ between laboratories and settings, it may be worth assessing the effects of different light, temperature, humidity conditions, and the time of the day on the ability of the device to show consistent data. Therefore, each laboratory should meet the minimum environmental conditions to set its own QC figures. Further studies may be required to assess whether the current FTD might allow estimating male mosquito competitiveness.

## Data Availability

The original contributions presented in the study are included in the article/Supplementary Material; further inquiries can be directed to the corresponding author.

## Supplementary Material

Supplementary Figure S1 Mean wing lengths of *Aedes albopictus* strains from different origins. Strains originated from Rimini, Italy; Valencia, Spain; and Guangzhou, China. Black bars indicate the median; the upper and lower limits of each box indicate the interquartile range. Each dot represents a value of measured wing length.

## References

[B1] Amos B. A. Ritchie S. A. Cardé R. T. (2020). Attraction versus Capture II: Efficiency of the BG-Sentinel Trap under Semifield Conditions and Characterizing Response Behaviors of Male *Aedes aegypti* (Diptera: Culicidae). J. Med. Entomology 57, 1539–1549. 10.1093/jme/tjaa065 PubMed Abstract | 10.1093/jme/tjaa065 | Google Scholar 32363393

[B2] Balestrino F. Puggioli A. Carrieri M. Bouyer J. Bellini R. (2017). Quality Control Methods for *Aedes albopictus* Sterile Male Production. PLoS Negl. Trop. Dis. 11, e0005881. 10.1371/journal.pntd.0005881 PubMed Abstract | 10.1371/journal.pntd.0005881 | Google Scholar 28892483 PMC5608434

[B3] Bellini R. Albieri A. Balestrino F. Carrieri M. Porretta D. Urbanelli S. (2010). Dispersal and Survival of *Aedes albopictus* (Diptera: Culicidae) Males in Italian Urban Areas and Significance for Sterile Insect Technique Application. Jnl. Med. Entom. 47, 1082–1091. 10.1603/me09154 PubMed Abstract | 10.1603/me09154 | Google Scholar 21175057

[B4] Benedict M. Q. Levine R. S. Hawley W. A. Lounibos L. P. (2007). Spread of the Tiger: Global Risk of Invasion by the MosquitoAedes Albopictus. Vector-Borne Zoonotic Dis. 7, 76–85. 10.1089/vbz.2006.0562 PubMed Abstract | 10.1089/vbz.2006.0562 | Google Scholar 17417960 PMC2212601

[B5] Benedict M. Robinson A. S. (2003). The First Releases of Transgenic Mosquitoes: An Argument for the Sterile Insect Technique. Trends Parasitol. 19, 349–355. 10.1016/s1471-4922(03)00144-2 PubMed Abstract | 10.1016/s1471-4922(03)00144-2 | Google Scholar 12901936

[B6] Bhatt S. Gething P. W. Brady O. J. Messina J. P. Farlow A. W. Moyes C. L. (2013). The Global Distribution and Burden of Dengue. Nature 496, 504–507. 10.1038/nature12060 PubMed Abstract | 10.1038/nature12060 | Google Scholar 23563266 PMC3651993

[B7] Bimbilé Somda N. S. Maïga H. Mamai W. Yamada H. Ali A. Konczal A. (2019). Insects to Feed Insects - Feeding Aedes Mosquitoes with Flies for Laboratory Rearing. Sci. Rep. 9, 11403–11413. 10.1038/s41598-019-47817-x PubMed Abstract | 10.1038/s41598-019-47817-x | Google Scholar 31388041 PMC6684809

[B8] Bolker B. (2018). GLMM FAQ: Testing for Overdispersion/Computing Overdispersion Factor. PubMed Abstract | Google Scholar

[B9] Bouyer J. Vreysen M. J. B. (2020). Yes, Irradiated Sterile Male Mosquitoes Can Be Sexually Competitive!. Trends Parasitol. 36, 877–880. 10.1016/j.pt.2020.09.005 PubMed Abstract | 10.1016/j.pt.2020.09.005 | Google Scholar 33036938

[B10] Bouyer J. Yamada H. Pereira R. Bourtzis K. Vreysen M. J. B. (2020). Phased Conditional Approach for Mosquito Management Using Sterile Insect Technique. Trends Parasitol. 36, 325–336. 10.1016/j.pt.2020.01.004 PubMed Abstract | 10.1016/j.pt.2020.01.004 | Google Scholar 32035818

[B11] Boyer S. Maillot L. Gouagna L.-C. Fontenille D. Chadee D. D. Lemperiere G. (2013). Diel Activity Patterns of MaleAedes Albopictusin the Laboratory. J. Am. Mosquito Control Assoc. 29, 74–77. 10.2987/12-6259r.1 PubMed Abstract | 10.2987/12-6259r.1 | Google Scholar 23687861

[B12] Bretz F. Hothorn T. Westfall P. (2016). Multiple Comparisons Using R. New York, NY: Chapman and Hall/CRC. Google Scholar

[B13] Briegel H. Knüsel I. Timmermann S. E. (2001). *Aedes aegypti*: Size, Reserves, Survival, and Flight Potential. J. Vector Ecol. 26, 21–31. PubMed Abstract | Google Scholar 11469181

[B14] Cabrera M. Jaffe K. (2007). An Aggregation Pheromone Modulates Lekking Behavior in the Vector Mosquito *Aedes aegypti* (Diptera: Culicidae). J. Am. Mosquito Control Assoc. 23, 1–10. 10.2987/8756-971x(2007)23[1:aapmlb]2.0.co;2 10.2987/8756-971x(2007)23[1:aapmlb]2.0.co;2 | Google Scholar 17536361

[B15] Chaves L. F. Chaves L. F. (2010). An Entomologist Guide to Demystify Pseudoreplication: Data Analysis of Field Studies with Design Constraints. J. Med. Entomol. 47, 291–298. 10.1093/jmedent/47.1.291 PubMed Abstract | 10.1093/jmedent/47.1.291 | Google Scholar 20496574

[B16] Crawley M. J. (2012). The R Book. New Delhi: John Wiley & Sons. Google Scholar

[B17] Crepeau T. N. Healy S. P. Bartlett-Healy K. Unlu I. Farajollahi A. Fonseca D. M. (2013). Effects of Biogents Sentinel Trap Field Placement on Capture Rates of Adult Asian Tiger Mosquitoes, *Aedes albopictus* . PloS one 8, e60524. 10.1371/journal.pone.0060524 PubMed Abstract | 10.1371/journal.pone.0060524 | Google Scholar 23555987 PMC3612070

[B18] Cui J. Li S. Zhao P. Zou F. (2013). Flight Capacity of Adult *Culex pipiens* Pallens (Diptera: Culicidae) in Relation to Gender and Day-Age. Jnl. Med. Entom. 50, 1055–1058. 10.1603/me12078 PubMed Abstract | 10.1603/me12078 | Google Scholar 24180110

[B19] Culbert N. J. Somda N. S. B. Hamidou M. Soma D. D. Caravantes S. Wallner T. (2020). A Rapid Quality Control Test to Foster the Development of the Sterile Insect Technique against *Anopheles Arabiensis* . Malar. J. 19, 44–10. 10.1186/s12936-020-3125-z PubMed Abstract | 10.1186/s12936-020-3125-z | Google Scholar 31973756 PMC6979282

[B20] Culbert N. J. Balestrino F. Dor A. Herranz G. S. Yamada H. Wallner T. (2018). A Rapid Quality Control Test to Foster the Development of Genetic Control in Mosquitoes. Sci. Rep. 8, 16179. 10.1038/s41598-018-34469-6 PubMed Abstract | 10.1038/s41598-018-34469-6 | Google Scholar 30385841 PMC6212531

[B21] Culbert N. J. Gilles J. R. L. Bouyer J. (2019). Investigating the Impact of Chilling Temperature on Male *Aedes aegypti* and *Aedes albopictus* Survival. PLoS One 14, e0221822. 10.1371/journal.pone.0221822 PubMed Abstract | 10.1371/journal.pone.0221822 | Google Scholar 31454400 PMC6711517

[B22] Dor A. Maggiani-Aguilera A. M. Valle-Mora J. Bond J. G. Marina C. F. Liedo P. (2020). Assessment of *Aedes aegypti* (Diptera: Culicidae) Males Flight Ability for SIT Application: Effect of Device Design, Duration of Test, and Male Age. J. Med. entomology 57, 824–829. 10.1093/jme/tjz226 PubMed Abstract | 10.1093/jme/tjz226 | Google Scholar 31808821

[B23] Dou Z. Madan A. Carlson J. S. Chung J. Spoleti T. Dimopoulos G. (2021). Acoustotactic Response of Mosquitoes in Untethered Flight to Incidental Sound. Sci. Rep. 11, 1–9. 10.1038/s41598-021-81456-5 PubMed Abstract | 10.1038/s41598-021-81456-5 | Google Scholar 33479423 PMC7820424

[B24] Drago A. Spano G. Faccioni G. Massella E. (2021). Olfactory Responsiveness of *Culex quinquefasciatus* and *Aedes albopictus* (Diptera: Culicidae): Interactions between Species, Age and Attractants. Eur. J. Entomology 118. 10.14411/eje.2021.018 10.14411/eje.2021.018 | Google Scholar

[B25] Dyck V. A. Hendrichs J. Robinson A. S. (Editors) (2021). Sterile Insect Technique: Principles and Practice in Area-wide Integrated Pest Management. 2nd ed. (Boca Raton, FL: CRC Press). 10.1201/9781003035572 10.1201/9781003035572

[B26] FAO/IAEA (2020). Guidelines for Mass Rearing *Aedes* Mosquitoes. Version 1.0. Available at: http://www-naweb.iaea.org/nafa/ipc/public/Guidelines-for-mass-rearingofAedes-osquitoes_v1.0.pdf . Google Scholar

[B27] FAO/IAEA (2017). Guidelines for Routine Colony Maintenance of *Aedes* Mosquito Species - Version 1.0. Available at: https://www.iaea.org/resources/manual/guidelines-for-routine-colony-maintenance-of-aedes-mosquito-species-version-10 (Accessed May 3, 2018). Google Scholar

[B28] Gómez-Simuta Y. Parker A. Cáceres C. Vreysen M. J. Yamada H. (2021). Characterization and Dose-Mapping of an X-Ray Blood Irradiator to Assess Application Potential for the Sterile Insect Technique (SIT). Appl. Radiat. Isotopes 176, 109859. 10.1016/j.apradiso.2021.109859 10.1016/j.apradiso.2021.109859 | Google Scholar 34284215

[B29] Hapairai L. K. Joseph H. Cheong Sang M. A. Melrose W. Ritchie S. A. Burkot T. R. (2013). Field Evaluation of Selected Traps and Lures for Monitoring the Filarial and Arbovirus Vector, *Aedes polynesiensis* (Diptera: Culicidae), in French Polynesia. Jnl. Med. Entom. 50, 731–739. 10.1603/me12270 PubMed Abstract | 10.1603/me12270 | Google Scholar 23926770

[B30] Harrison X. A. (2015). A Comparison of Observation-Level Random Effect and Beta-Binomial Models for Modelling Overdispersion in Binomial Data in Ecology & Evolution. PeerJ 3, e1114. 10.7717/peerj.1114 PubMed Abstract | 10.7717/peerj.1114 | Google Scholar 26244118 PMC4517959

[B31] Iyaloo D. P. Facknath S. Bheecarry A. (2020). A Mark-Release-Recapture Experiment with Radio-Sterilised *Aedes albopictus* Males as Part of a Sterile Insect Technique Programme against the Vector Mosquito in Panchvati, Mauritius. Afr. Entomol. 28, 187–191. 10.4001/003.028.0187 10.4001/003.028.0187 | Google Scholar

[B32] Johnson B. G. Rowley W. A. (1972). Age-related Ultrastructural Changes in the Flight Muscle of the Mosquito, *Culex Tarsalis* . J. Insect Physiology 18, 2375–2389. 10.1016/0022-1910(72)90182-5 PubMed Abstract | 10.1016/0022-1910(72)90182-5 | Google Scholar 4641410

[B33] Kéry M. Hatfield J. S. (2003). Normality of Raw Data in General Linear Models: the Most Widespread Myth in Statistics. Bull. Ecol. Soc. Am. 84, 92–94. 10.1890/0012-9623(2003)84[92:NORDIG]2.0.CO;2 10.1890/0012-9623(2003)84[92:NORDIG]2.0.CO;2 | Google Scholar

[B34] Knipling E. F. Laven H. Craig G. B. Pal R. Kitzmiller J. B. Smith C. N. (1968). Genetic Control of Insects of Public Health Importance. Bull. World Health Organ 38, 421–438. PubMed Abstract | Google Scholar 5302334 PMC2554477

[B35] Krockel U. Rose A. Eiras A. E. Geier M. (2006). New Tools for Surveillance of Adult Yellow Fever Mosquitoes: Comparison of Trap Catches with Human Landing Rates in an Urban Environment. J. Am. Mosq. Control Assoc. 22, 229–238. 10.2987/8756-971X(2006)22[229:NTFSOA]2.0.CO;2 PubMed Abstract | 10.2987/8756-971X(2006)22[229:NTFSOA]2.0.CO;2 | Google Scholar 17019768

[B36] Lebon C. Soupapoule K. Wilkinson D. A. Le Goff G. Damiens D. Gouagna L. C. (2018). Laboratory Evaluation of the Effects of Sterilizing Doses of γ-rays from Caesium-137 Source on the Daily Flight Activity and Flight Performance of *Aedes albopictus* Males. PLoS ONE 13, e0202236. 10.1371/journal.pone.0202236 PubMed Abstract | 10.1371/journal.pone.0202236 | Google Scholar 30107004 PMC6091941

[B37] Lenth R. (2020). Emmeans: Estimated Marginal Means, Aka Least-Squares Means. R Package Version 1.4.6. Available at: https://CRAN.R-project.org/package=emmeans (Accessed on October 10, 2020). Google Scholar

[B38] Levy-Blitchtein S. del Valle-Mendoza J. (2016). Zika Virus Is Arriving at the American Continent. Asian Pac. J. Trop. Med. 9, 1019–1021. 10.1016/j.apjtm.2016.07.030 PubMed Abstract | 10.1016/j.apjtm.2016.07.030 | Google Scholar 27794382

[B39] Li Y. Zhang M. Wang X. Zheng X. Hu Z. Xi Z. (2021). Quality Control of Long-Term Mass-Reared *Aedes albopictus* for Population Suppression. J. Pest Sci. 94, 1531–1542. 10.1007/s10340-021-01340-z 10.1007/s10340-021-01340-z | Google Scholar

[B40] Maïga H. Mamai W. Somda N. S. B. Konczal A. Wallner T. Herranz G. S. (2019). Reducing the Cost and Assessing the Performance of a Novel Adult Mass-Rearing Cage for the Dengue, Chikungunya, Yellow Fever and Zika Vector, *Aedes aegypti* (Linnaeus). PLoS neglected Trop. Dis. 13, e0007775. 10.1371/journal.pntd.0007775 10.1371/journal.pntd.0007775 | Google Scholar PMC6779276 31553724

[B41] Mamai W. Bimbilé Somda N. S. Maiga H. Konczal A. Wallner T. Bakhoum M. T. (2019). Black Soldier Fly (*Hermetia Illucens*) Larvae Powder as a Larval Diet Ingredient for Mass-Rearing *Aedes* Mosquitoes. Parasite 26, 57. 10.1051/parasite/2019059 PubMed Abstract | 10.1051/parasite/2019059 | Google Scholar 31535969 PMC6752115

[B42] Moyes C. L. Vontas J. Martins A. J. Ng L. C. Koou S. Y. Dusfour I. (2017). Contemporary Status of Insecticide Resistance in the Major *Aedes* Vectors of Arboviruses Infecting Humans. PLoS Negl. Trop. Dis. 11, e0005625. 10.1371/journal.pntd.0005625 PubMed Abstract | 10.1371/journal.pntd.0005625 | Google Scholar 28727779 PMC5518996

[B43] Nasci R. S. (1990). Relationship of Wing Length to Adult Dry Weight in Several Mosquito Species (Diptera: Culicidae). J. Med. Entomology 27, 716–719. 10.1093/jmedent/27.4.716 PubMed Abstract | 10.1093/jmedent/27.4.716 | Google Scholar 2388250

[B44] Oliva C. F. Jacquet M. Gilles J. Lemperiere G. Maquart P.-O. Quilici S. (2012). The Sterile Insect Technique for Controlling Populations of *Aedes albopictus* (Diptera: Culicidae) on Reunion Island: Mating Vigour of Sterilized Males. PLoS One 7, e49414. 10.1371/journal.pone.0049414 PubMed Abstract | 10.1371/journal.pone.0049414 | Google Scholar 23185329 PMC3504010

[B45] Parker A. G. Vreysen M. J. B. Bouyer J. Calkins C. O. (2021). “Sterile Insect Quality Control/assurance,” in Sterile Insect Technique. Principles and Practice in Area-wide Integrated Pest Management. Editors Dyck V. A. Hendrichs J. P. Robinson A. S. (Boca Raton, FL: CRC Press), 399–440. Available at: https://www.taylorfrancis.com/chapters/sterile-insect-quality-control-assurance-parker-vreysen-bouyer-calkins/e/10.1201/9781003035572-12 . 10.1201/9781003035572-12 10.1201/9781003035572-12 | Google Scholar

[B46] Pitts R. J. Mozūraitis R. Gauvin-Bialecki A. Lempérière G. (2013). The Roles of Kairomones, Synomones and Pheromones in the Chemically-Mediated Behaviour of Male Mosquitoes. Acta Trop. 132 (Suppl. l), S26–S34. 10.1016/j.actatropica.2013.09.005 PubMed Abstract | 10.1016/j.actatropica.2013.09.005 | Google Scholar 24055544

[B47] Pombi M. Jacobs F. Verhulst N. O. Caputo B. della Torre A. Takken W. (2014). Field Evaluation of a Novel Synthetic Odour Blend and of the Synergistic Role of Carbon Dioxide for Sampling Host-Seeking *Aedes albopictus* Adults in Rome, Italy. Parasit. Vectors 7, 580–585. 10.1186/s13071-014-0580-9 PubMed Abstract | 10.1186/s13071-014-0580-9 | Google Scholar 25499569 PMC4271472

[B48] Rowley W. A. Graham C. L. (1968). The Effect of Age on the Flight Performance of Female *Aedes aegypti* Mosquitoes. J. insect physiology 14, 719–728. 10.1016/0022-1910(68)90230-8 10.1016/0022-1910(68)90230-8 | Google Scholar 5649625

[B49] Rowley W. A. Graham C. L. Williams R. E. (1968). A Flight Mill System for the Laboratory Study of Mosquito Flight. Ann. Entomological Soc. Am. 61, 1507–1514. 10.1093/aesa/61.6.1507 10.1093/aesa/61.6.1507 | Google Scholar

[B50] Shelly T. E. Edu J. Nishimoto J. (2010). Chilling and Flight Ability and Mating Competitiveness of Sterile Males of the Mediterranean Fruit Fly. J. Appl. Entomology 134. 10.1111/j.1439-0418.2010.01532.x 10.1111/j.1439-0418.2010.01532.x | Google Scholar

[B51] Staunton K. M. Leiva D. Cruz A. Goi J. Arisqueta C. Liu J. (2021). Outcomes from International Field Trials with Male *Aedes* Sound Traps: Frequency-dependent Effectiveness in Capturing Target Species in Relation to Bycatch Abundance. PLoS Negl. Trop. Dis. 15, e0009061. 10.1371/journal.pntd.0009061 PubMed Abstract | 10.1371/journal.pntd.0009061 | Google Scholar 33630829 PMC7906331

[B52] Swan T. Russell T. L. Burkot T. R. Liu J. Ritchie S. A. Staunton K. M. (2021). The Effect of Sound Lure Frequency and Habitat Type on Male *Aedes albopictus* (Diptera: Culicidae) Capture Rates with the Male *Aedes* Sound Trap. J. Med. entomology 58, 708–716. 10.1093/jme/tjaa242 10.1093/jme/tjaa242 | Google Scholar PMC7954095 33179740

[B53] Visser T. M. De Cock M. P. Hiwat H. Wongsokarijo M. Verhulst N. O. Koenraadt C. J. (2020). Optimisation and Field Validation of Odour-Baited Traps for Surveillance of *Aedes aegypti* Adults in Paramaribo, Suriname. Parasites vectors 13, 1–14. 10.1186/s13071-020-4001-y PubMed Abstract | 10.1186/s13071-020-4001-y | Google Scholar 32143711 PMC7059684

[B54] Vreysen M. J. B. Abd-Alla A. M. M. Bourtzis K. Bouyer J. Caceres C. de Beer C. (2021). The Insect Pest Control Laboratory of the Joint FAO/IAEA Programme: Ten Years (2010-2020) of Research and Development, Achievements and Challenges in Support of the Sterile Insect Technique. Insects 12, 346. 10.3390/insects12040346 PubMed Abstract | 10.3390/insects12040346 | Google Scholar 33924539 PMC8070182

[B55] Wehmann H. N. Engels T. Lehmann F. O. (2022). Flight Activity and Age Cause Wing Damage in House Flies. J. Exp. Biol. 225, jeb242872. 10.1242/jeb.242872 PubMed Abstract | 10.1242/jeb.242872 | Google Scholar 34904650

[B56] WHO and IAEA (2020). TDR | Guidance Framework for Testing the Sterile Insect Technique as a Vector Control Tool against Aedes-Borne Diseases. Geneva: WHO. Available at: https://www.who.int/publications/i/item/9789240002371 . Google Scholar

[B57] Wigglesworth V. B. (1949). The Utilization of Reserve Substances in Drosophila during Flight. J. Exp. Biol. 26, 150–163. 10.1242/jeb.26.2.150 PubMed Abstract | 10.1242/jeb.26.2.150 | Google Scholar 15395188

[B58] Williams C. M. Barness L. A. Sawyer W. H. (1943). The Utilization of Glycogen by Flies during Flight and Some Aspects of the Physiological Ageing of Drosophila. Biol. Bull. 84, 263–272. 10.2307/1538009 10.2307/1538009 | Google Scholar

