# Supplementary material for: Standardization of the FAO/IAEA Flight Test for Quality Control of Sterile Mosquitoes
Source: Front Bioeng Biotechnol. 2022 Jul 18;10:876675. doi: 10.3389/fbioe.2022.876675 (PMC9341283; doi:10.3389/fbioe.2022.876675)
Supplement: Supplementary file 1 [file DataSheet1.zip › Supplementary Materials/supp mat. 13 and 16 word versions/Supplementary Material S13..docx]

**Standardization of the FAO/IAEA flight test for quality control of sterile mosquitoes**

Hamidou Maïga, Deng Lu, Wadaka Mamai, Nanwintoum Séverin Bimbilé Somda, Thomas Wallner, Mame Thierno Bakhoum, Odet Bueno Masso, Claudia Martina, Simran Singh Kotla, Hanano Yamada, Gustavo Salvador Herranz, Rafael Argiles Herrero, Chee Seng Chong, Cheong Huat Tan, Jeremy Bouyer

**Supplementary Material S13.** Script of the Flight ability test video

<https://www.dropbox.com/s/22tks0hhp31qokq/Flight%20test%20device%20instructional%20video%20cut2%20with%20sub%20and%20music.mp4?dl=0>

**0:00-0:06 “**The flight ability of mosquitoes can be measured using a Flight Test Device, as developed by the Insect Pest Control Subprogramme.”

**0:19-0:28 “**The Flight Test Device consists of six separate parts:

- a base plate;
- a transparent containment box with an open top and a circular netted opening;
- a top cover;
- the flight tube itself, composed of 40 individual tube’s each 25 cm high with an inside diameter of 8 mm placed inside a large containment tube;
- a 12V fan with an air flow of 0.218m³/min, am acoustic noise of 20.6dB and a rated speed of 6000rpm;
- and finally, a rubber base ring with a square of netting.”

**0:29-0:39 “**To assemble the Flight Test Device, begin by placing the base plate inside the rectangular containment box. The base should be placed with the metal rods facing downwards so that there is a space between the bottom of the containment box and the base plate.

**0:39-0:51 “**Next, cover the rubber base ring with the square of netting and fit it to the bottom of the large containment tube. Make sure the rubber ring fits the containment tube tightly, but do not to cover the small, 1 cm hole at the bottom of the tube yet.”

**0:52-0:57 “**Move the tube inside the rectangular containment box and cover the latter with the top cover.”

**0:59-1:06 “**Put 2-3 small pellets of BG lure from Biogents, Regensburg, Germany in the decoy cap on the top cover.”

**1:07-1:13 “**Place the 12V fan directly on the decoy cap facing downwards so that air is blown into the containment box.”

**1:17-1:24 “**Switch on the fan for all Flight Test Devices. Gently aspirate 100 male mosquitoes of the same age with a manual aspirator.”

**1:28-1:37** “Release them via the small hole at the bottom into the large containment tube of the Flight Test Device. Use the containment box’s netted opening to ensure mosquitoes do not escape the Flight Test Device.”

**1:37-1:45** “Once all the mosquitoes are inside the containment tube, push the rubber base ring and netting upwards to cover the small hole through which mosquitoes were introduced by attaching the containment tube to the base plate. Repeat this procedure for at least 5 replicates per treatment that can be run in parallel.”

**1:48-2:14 “**Confined within a small volume, the instinct of mosquitoes is to fly upwards, through one of the 40 flight tubes, and out into the large, containment tube.”

**2:19-2:25** “After two hours, cover the top of the containment tube with a petri dish of 9 cm of diameter and turn off all fans. At this point, the experiment is considered as complete.”

**2:26-2:32** “Slowly remove the containment tube through the net of the large circular opening in the containment box.”

**2:32-2:36** “When the tube is half-way out, be careful to hold the petri dish covering the top of the tube in place, to avoid any escapees.”

**2:36-2:41** “The mosquitoes remaining in the tube are considered as ‘Non-fliers’ whereas the ones remaining in the containment box are considered as ‘fliers” to calculate the flight rate.”

***Important:*** *to avoid additional stress during mosquito aspiration, calibrate the mouth aspirator by marking a line corresponding to a level of known number of mosquitoes. For example, a line can, be marked onto the bottom of the aspirator for about 100 males and can be routinely used for quality control flight test operations.*
