# Supplementary material for: Standardization of the FAO/IAEA Flight Test for Quality Control of Sterile Mosquitoes
Source: Front Bioeng Biotechnol. 2022 Jul 18;10:876675. doi: 10.3389/fbioe.2022.876675 (PMC9341283; doi:10.3389/fbioe.2022.876675)
Supplement: Supplementary file 1 [file DataSheet1.zip › Supplementary Materials/supp mat. 13 and 16 word versions/Supplementary Material S16. Experiments_Effects of shadow and dark cloth as attractant.docx]

**Standardization of the FAO/IAEA flight test for quality control of sterile mosquitoes**

Hamidou Maïga, Deng Lu, Wadaka Mamai, Nanwintoum Séverin Bimbilé Somda, Thomas Wallner, Mame Thierno Bakhoum, Odet Bueno Masso, Claudia Martina, Simran Singh Kotla, Hanano Yamada, Gustavo Salvador Herranz, Rafael Argiles Herrero, Chee Seng Chong, Cheong Huat Tan, Jeremy Bouyer

**Supplementary Material S16** **_ Additional Experiments**

**2.6. Effects of internal tube color and addition of lure and fan on escape rate of *Aedes aegypti* and *Aedes albopictus***

*Effects of shadow on male escape rate*

To confirm that pink silicon in each inner tube end could impact the escape rates because of the presence of a shadow, we introduced a 2×29 cm black piece of paper. The paper was rolled around each end of the tube to form a shadow around the top and bottom of the transparent inner tube. Male *Ae. albopictus* mosquitoes’ ability to fly through three shadowed FTDs was compared to that of mosquitoes flying through three FTDs with transparent and pink tubes (**Figure 1)**.


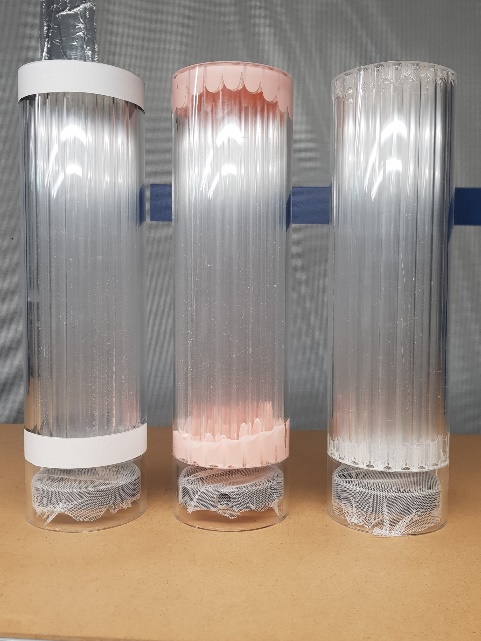


**Figure 1.** (A) The paper 2×29cm black piece of paper (on one side) was rolled around each end of the tube to form a shadow around the top and bottom of the transparent/white inner tube ; (B) Pink-colored inner tube; (C) transparent/white inner tube

*Effects of black cloth as an attractant against the lure and fan on male escape rate*

To assess whether using a piece of black cloth as an attractant would impact the escape rate from the pink-colored FTD as compared to the combination of lure and fan without the use of cloth, an 8cm-diameter black cloth (corresponding to the diameter of the inner tube of the FTD) was placed on top of the FTD (**Figure 2**). Four FTDs of each of the two treatments (pink FTD with lure and fan, pink FTD with 8cm black cloth) were tested with two- to three-day-old male *Ae. aegypti* and *Ae. albopictus* mosquitoes.


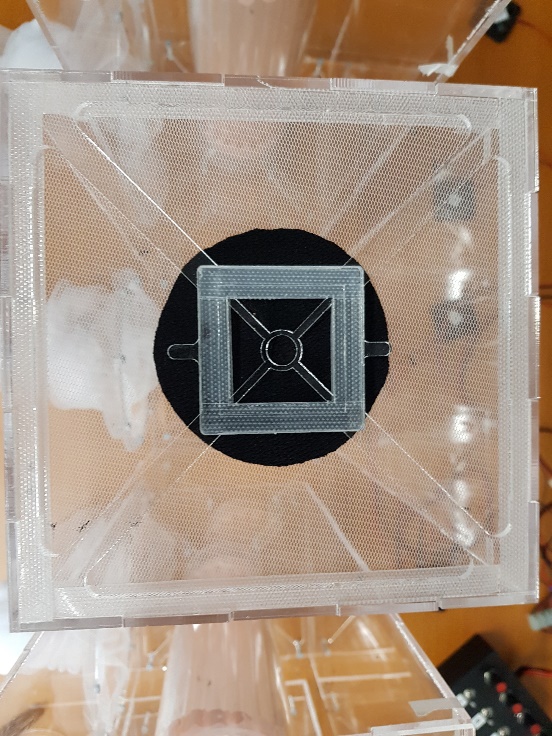


**Figure 2**. An 8cm-diameter black cloth (corresponding to the diameter of the inner tube of the FTD) as an attractant was placed on the top cover of the FTD

**Results**

*Effects of shadow on male escape rate*

When the effect of shadow was tested to confirm whether or not pink silicon in each end of the inner tube would impact the escape rates, it was shown that the treatment had a significant effect (χ^2^ = 39.6, df = 2, *p* < 0.001, **Figure 3).** A pairwise comparison of means showed that fewer male *Ae. albopictus* (Rimini strain) mosquitoes escaped from the transparent tube with added shade (*p* < 0.0002) and from the pink colored tube (*p* < 0.0001) as compared to the transparent tube, whereas a similar number of escapees were observed between the pink and shadowed tube (*p* = 0.07).


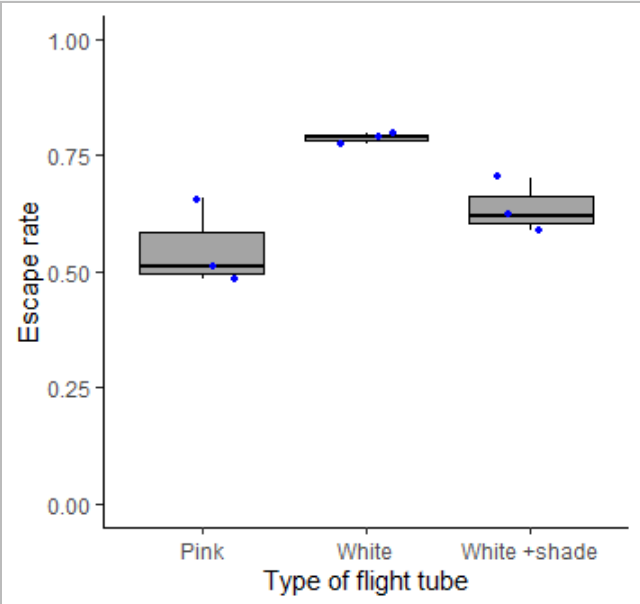


**FIGURE 3.** Male *Aedes albopictus* mosquitoes escape rates according to the type of flight test tubes. Three tubes including pink, transparent and transparent shadowed around the inner tube at the top and the bottom ends, were tested.

*Effect of black cloth as attractant against lure and fan on male escape rate*

The 8cm-diameter piece of black cloth placed on top of the FTD showed a lower escape rate from the pink colored FTD as compared to the pink colored FTD with fan and lure (χ^2^ = 4.78, df = 1, *p* = 0.028; **Figure 4).** There was a significant difference between species with male *Ae. aegypti* escaping more than *Ae. albopictus* from the pink colored FTD (χ^2^ = 16.17, df = 1, *p* < 0.001; **Figure 4).**


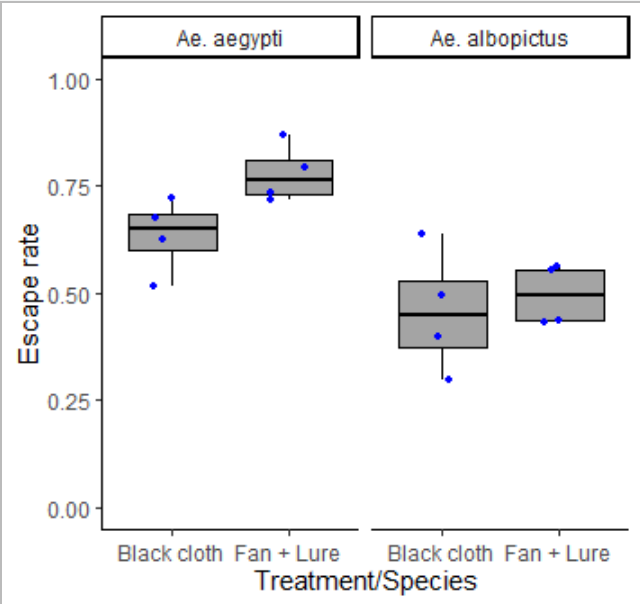


**FIGURE 4.** Effect of black cloth as attractant on male *Aedes aegypti* and *Aedes albopictus* mosquito, escape rates from the pink colored FTD as compared to fan and lure.
