# Supplementary material for: Standardization of the FAO/IAEA Flight Test for Quality Control of Sterile Mosquitoes
Source: Front Bioeng Biotechnol. 2022 Jul 18;10:876675. doi: 10.3389/fbioe.2022.876675 (PMC9341283; doi:10.3389/fbioe.2022.876675)
Supplement: Supplementary file 1 [file DataSheet1.zip › Supplementary Materials/Supplementary Material S1..pptx]

## Slide 1
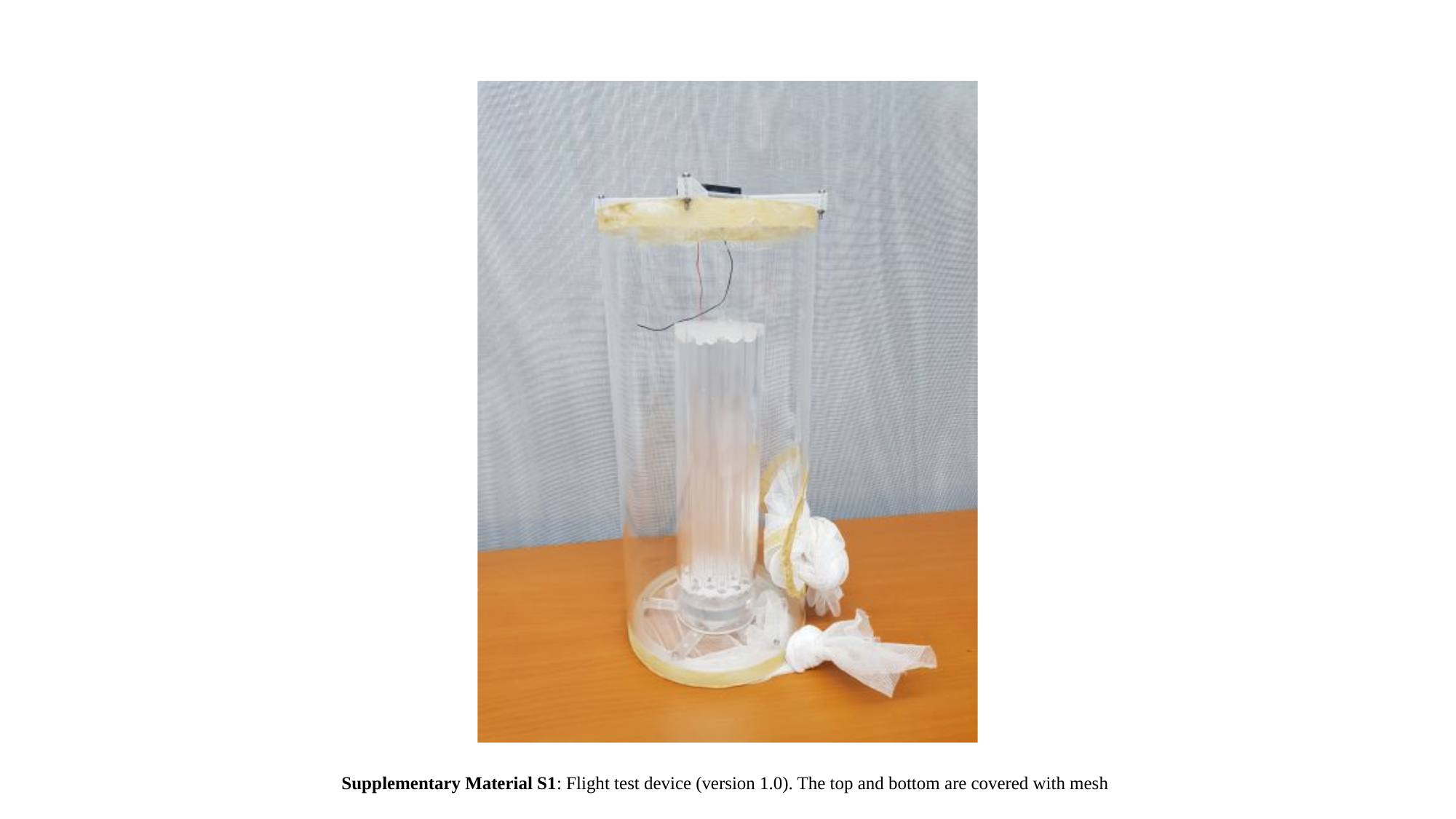

Supplementary Material S1: Flight test device (version 1.0). The top and bottom are covered with mesh
