# Supplementary material for: Standardization of the FAO/IAEA Flight Test for Quality Control of Sterile Mosquitoes
Source: Front Bioeng Biotechnol. 2022 Jul 18;10:876675. doi: 10.3389/fbioe.2022.876675 (PMC9341283; doi:10.3389/fbioe.2022.876675)
Supplement: Supplementary file 1 [file DataSheet1.zip › Supplementary Materials/Supplementary Material S11. Top Cover Parts 1_1.pdf]

4.1

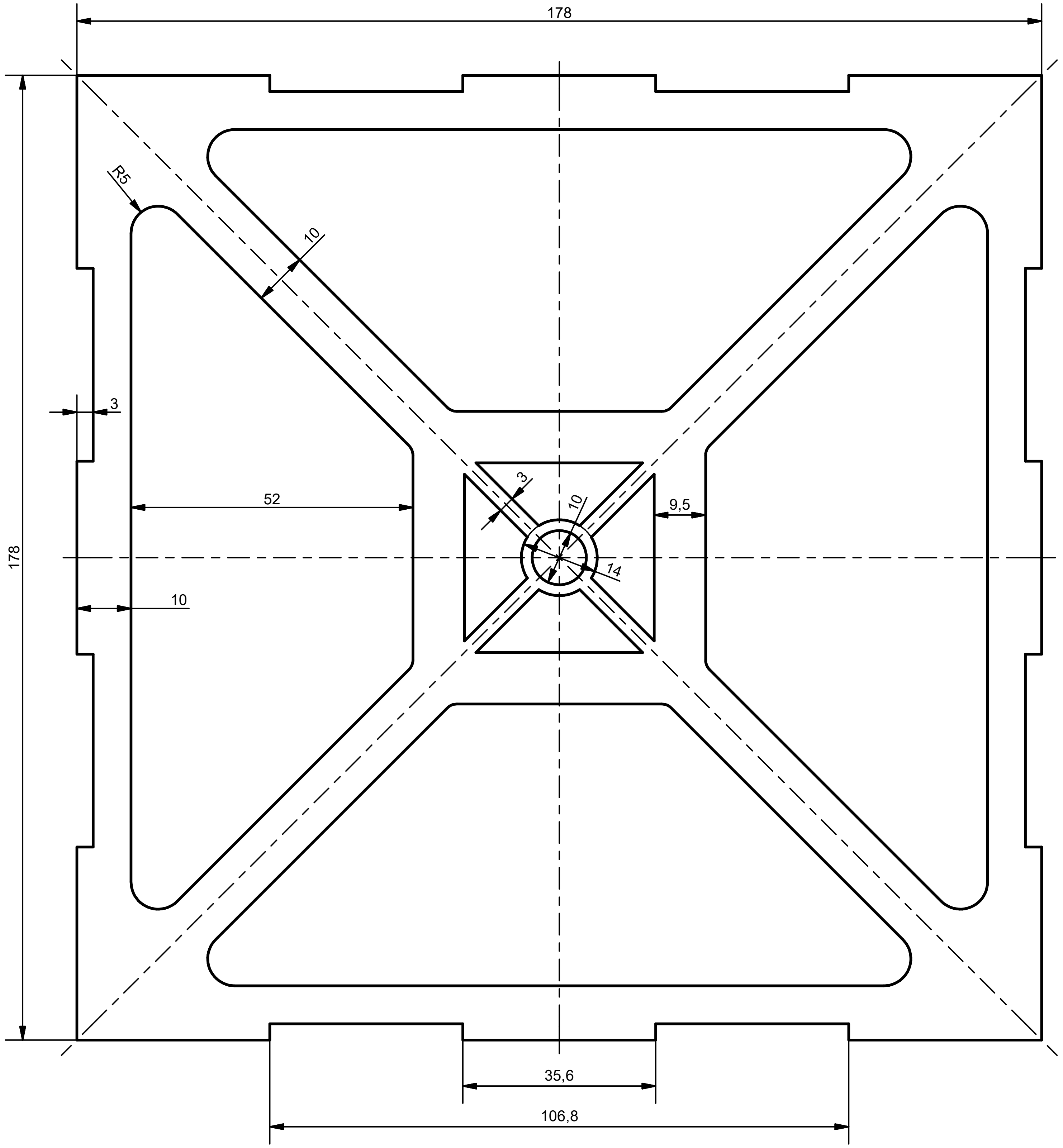

4.2

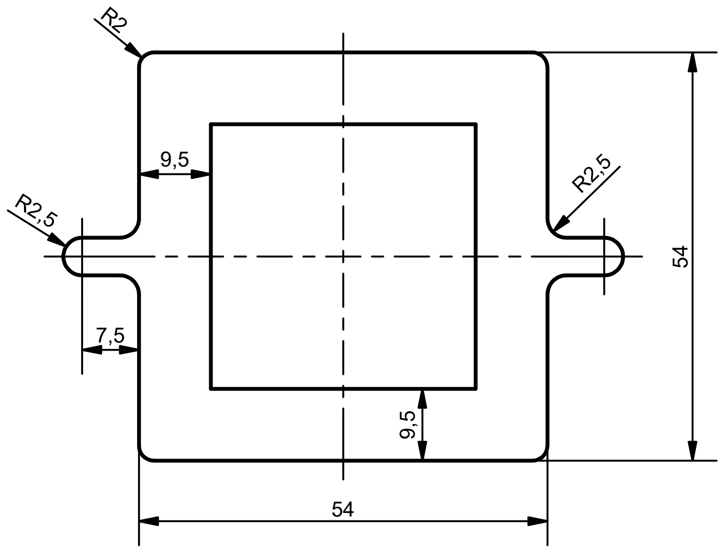

4.3

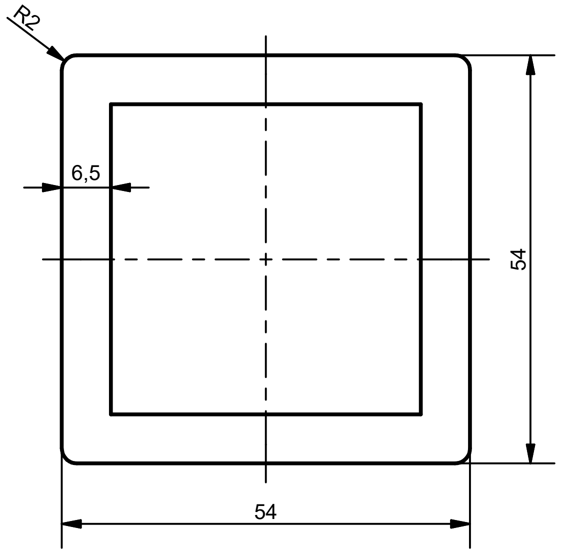

3mm transparent PMMA

	Name	Date
Designed	G. Salvador-Herranz	2020/06/22
Revised	R. Argilés	2020/06/22

Joint FAO/IAEA Programme

Insect Pest Control Section

Scale: 1:1 mm

Flight Ability Test Device

Top Cover - Parts 1/1

Number: FATD_V1

Sheet: 11/11
