# Supplementary material for: Standardization of the FAO/IAEA Flight Test for Quality Control of Sterile Mosquitoes
Source: Front Bioeng Biotechnol. 2022 Jul 18;10:876675. doi: 10.3389/fbioe.2022.876675 (PMC9341283; doi:10.3389/fbioe.2022.876675)
Supplement: Supplementary file 1 [file DataSheet1.zip › Supplementary Materials/Supplementary Material S12. Top Cover Assembly.pdf]

4

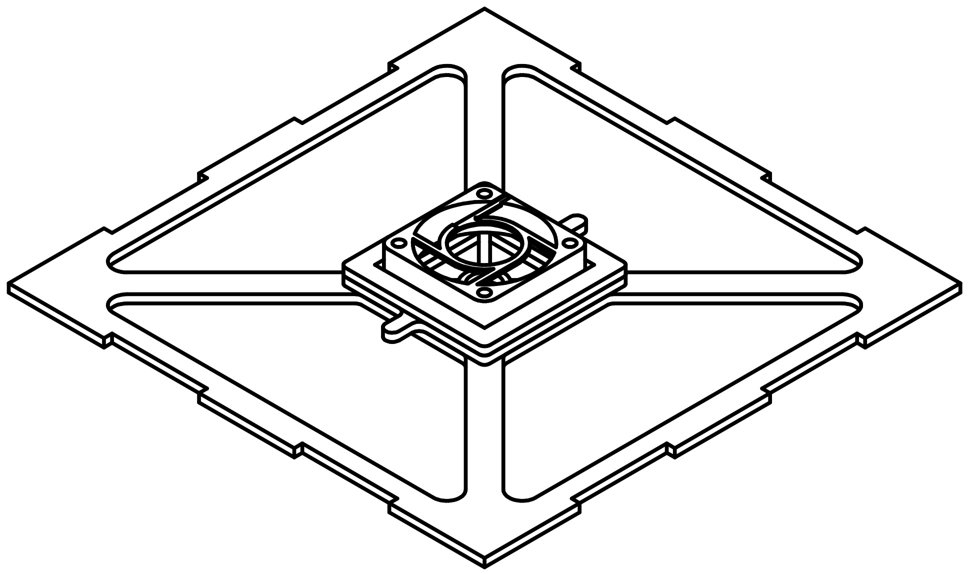

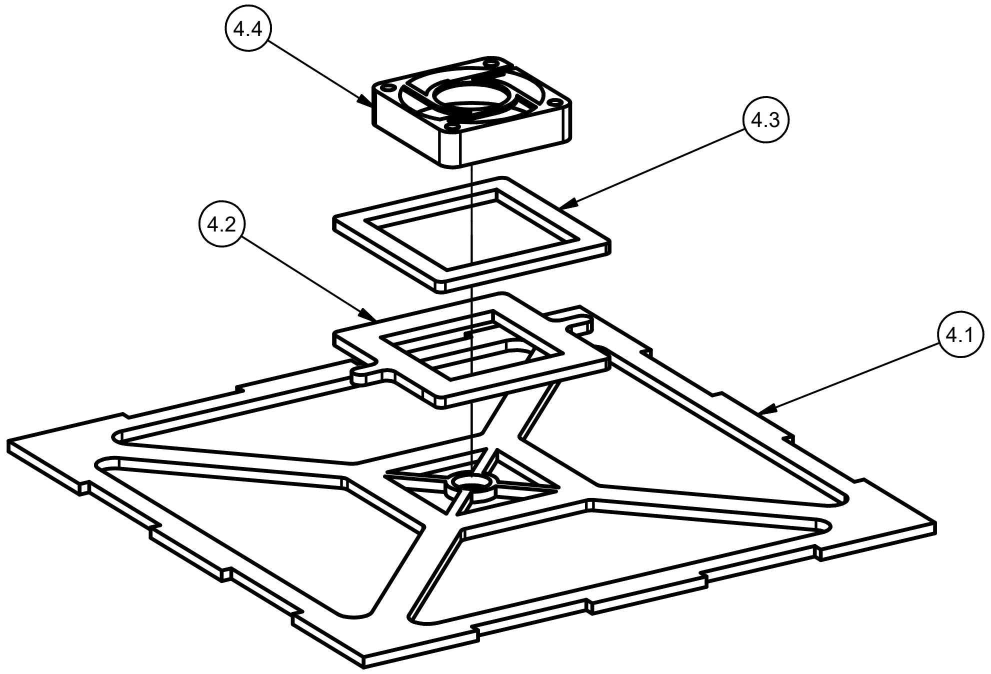

4.4	1	Fan	40x40mm - 12 v DC - 11.9 m3/hour
4.3	1	Top frame	3mm transparent PMMA
4.2	1	Bottom frame	3mm transparent PMMA
4.1	1	Support base	3mm transparent PMMA
Item	Quantity	Part	Description

	Name	Date
Designed	G. Salvador-Herranz	2020/06/22
Revised	R. Argilés	2020/06/22

Joint FAO/IAEA Programme

Insect Pest Control Section

Scale: mm

Flight Ability Test Device

Top Cover - Assembly

Number: FATD_V1

Sheet: 10/11
