# Supplementary material for: Standardization of the FAO/IAEA Flight Test for Quality Control of Sterile Mosquitoes
Source: Front Bioeng Biotechnol. 2022 Jul 18;10:876675. doi: 10.3389/fbioe.2022.876675 (PMC9341283; doi:10.3389/fbioe.2022.876675)
Supplement: Supplementary file 1 [file DataSheet1.zip › Supplementary Materials/Supplementary Material S5. Outer Container Assembly Parts 1_2.pdf]

## 1.1

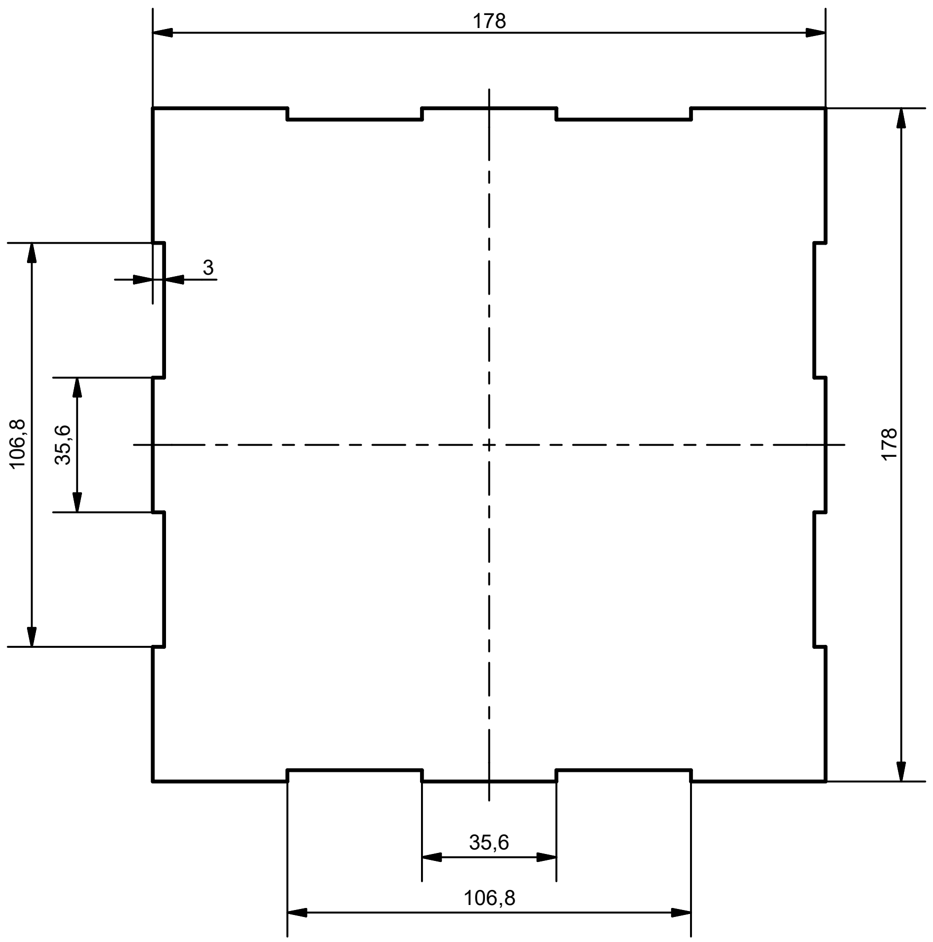


## 1.5

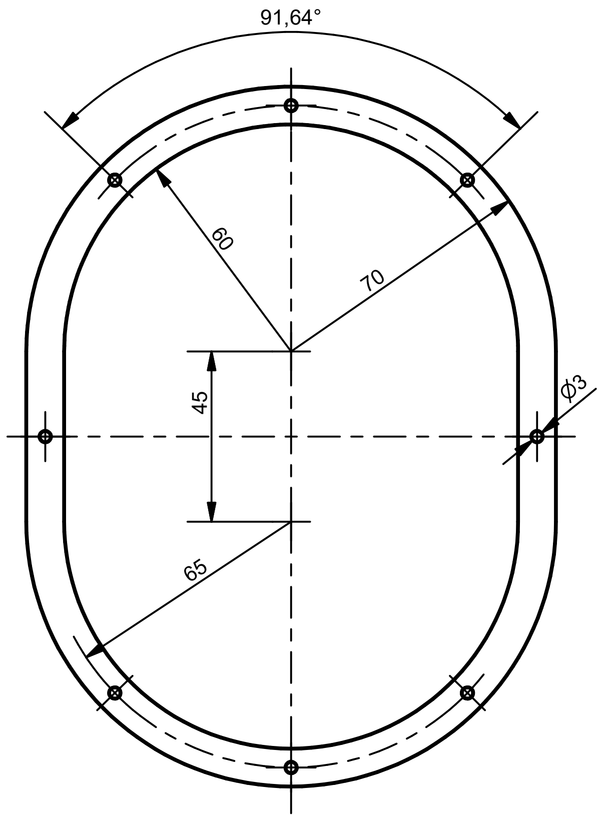


3mm transparent PMMA

| | Name | Date | | |
|---|---|---|---|---|
| Designed | G. Salvador-Herranz | 2020/06/22 | | Joint FAO/IAEA Programme Insect Pest Control Section |
| Revised | R. Argilés | 2020/06/22 | | |
| Scale 1:2 mm | Flight Ability Test Device | | | Number FATD_V1 |
| | Outer Container Assembly - Parts 1/2 | | | Sheet 4/11 |
