# Supplementary material for: Standardization of the FAO/IAEA Flight Test for Quality Control of Sterile Mosquitoes
Source: Front Bioeng Biotechnol. 2022 Jul 18;10:876675. doi: 10.3389/fbioe.2022.876675 (PMC9341283; doi:10.3389/fbioe.2022.876675)
Supplement: Supplementary file 1 [file DataSheet1.zip › Supplementary Materials/Supplementary Material S6. Outer Container Assembly Parts 2_2.pdf]

1.2

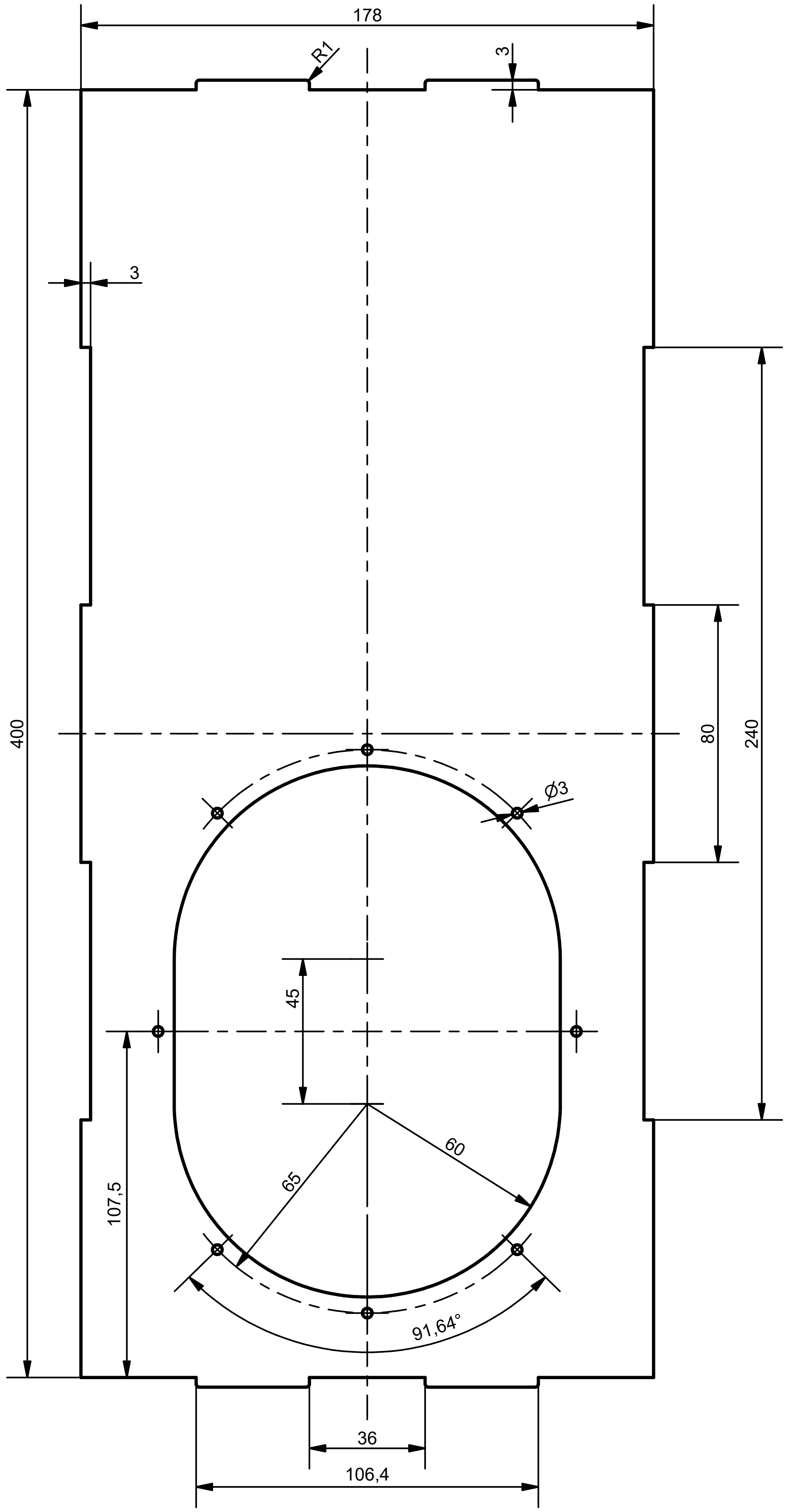

1.3

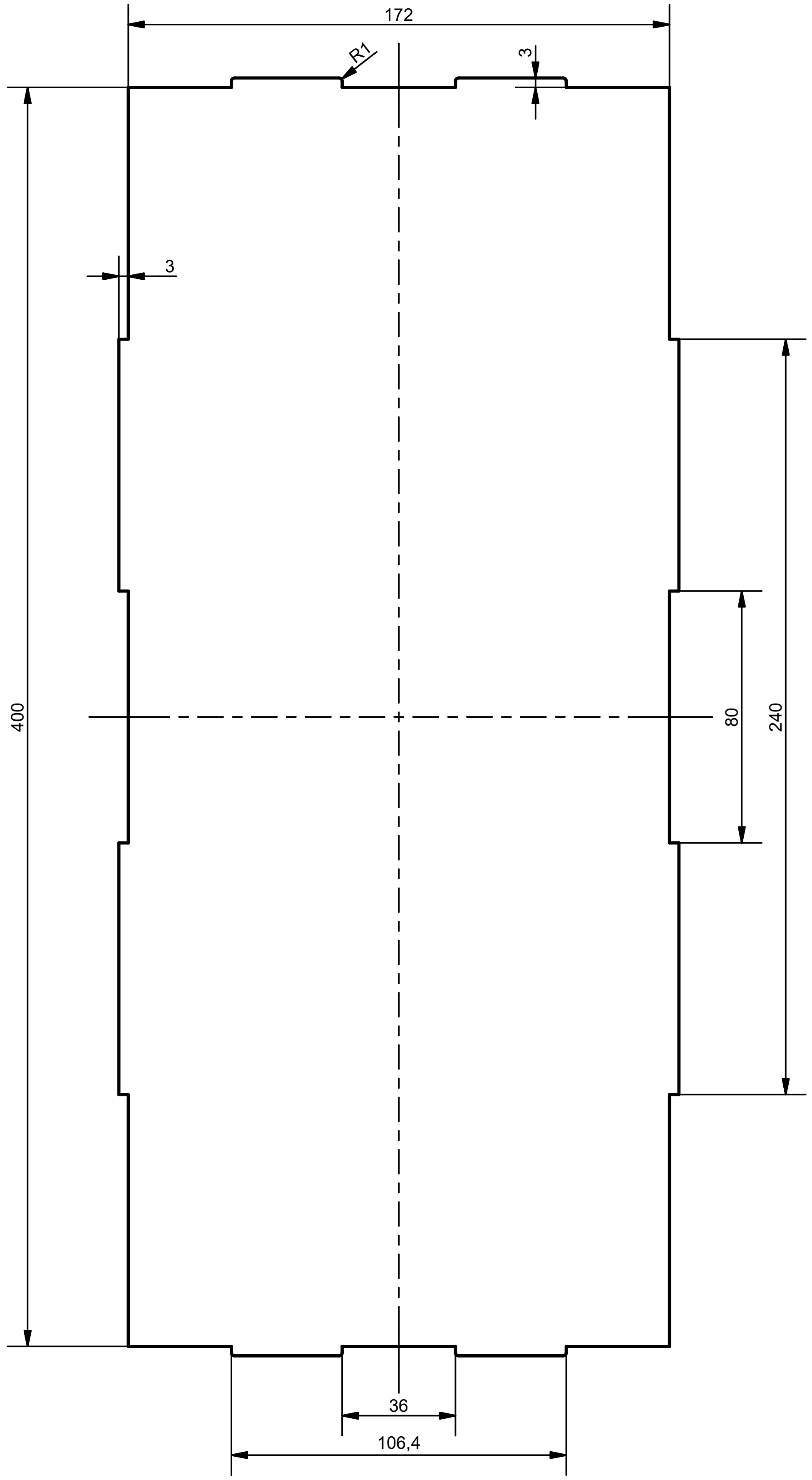

1.4

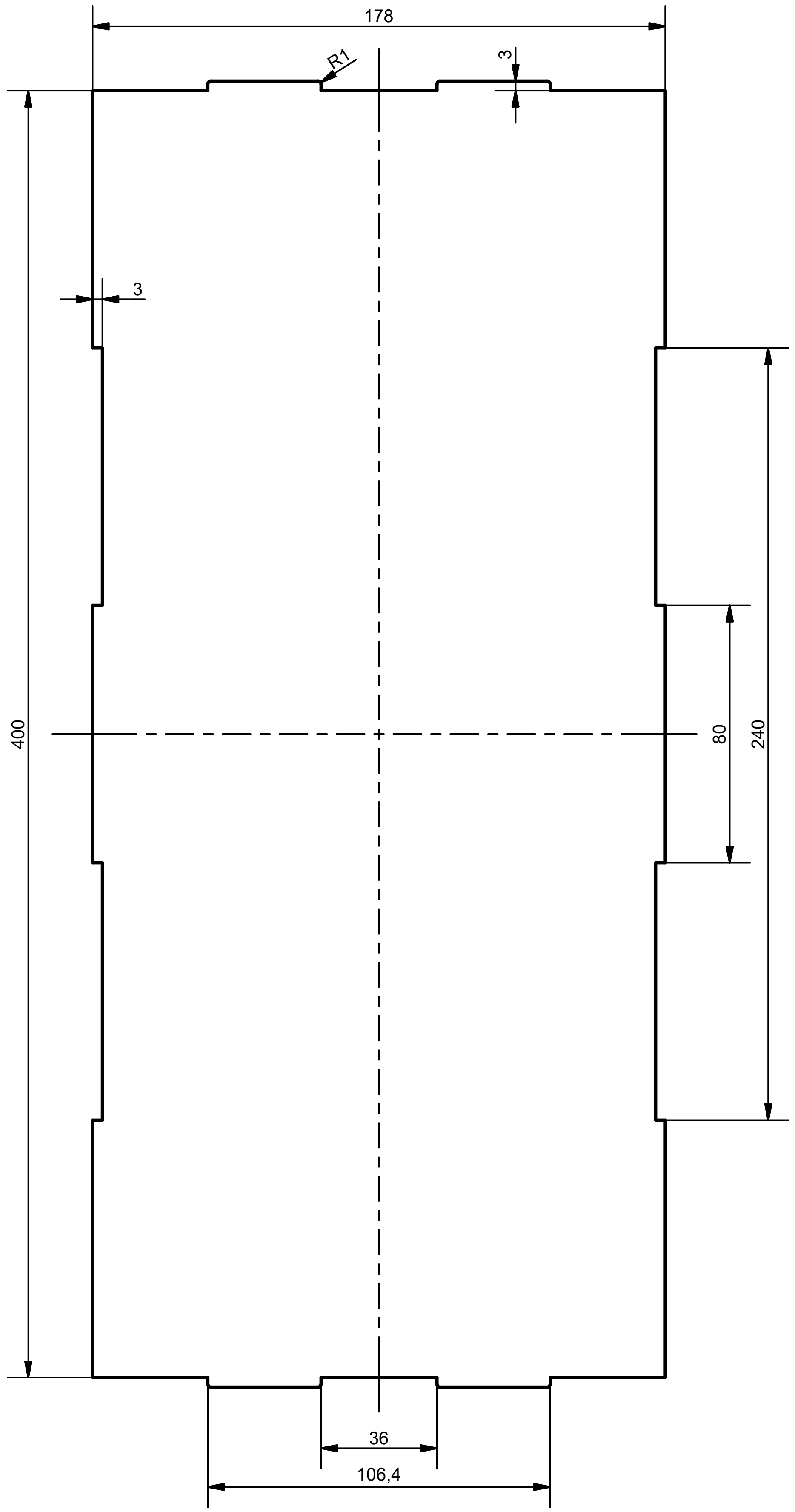

3mm transparent PMMA

	Name	Date		
Designed	G. Salvador-Herranz	2020/06/22		Joint FAO/IAEA Programme — Insect Pest Control Section
Revised	R. Argilés	2020/06/22		
Scale 1:2 mm	Flight Ability Test Device<br>Outer Container Assembly - Parts 2/2			Number FATD_V1<br>Sheet 5/11
