# Supplementary material for: Standardization of the FAO/IAEA Flight Test for Quality Control of Sterile Mosquitoes
Source: Front Bioeng Biotechnol. 2022 Jul 18;10:876675. doi: 10.3389/fbioe.2022.876675 (PMC9341283; doi:10.3389/fbioe.2022.876675)
Supplement: Supplementary file 1 [file DataSheet1.zip › Supplementary Materials/Supplementary Material S7. Inner flight tube holder.pdf]

2

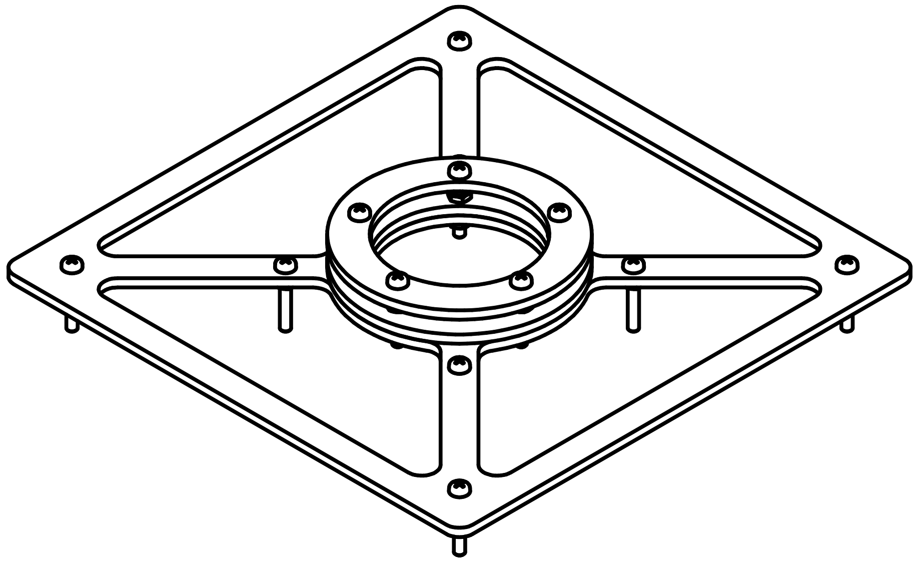

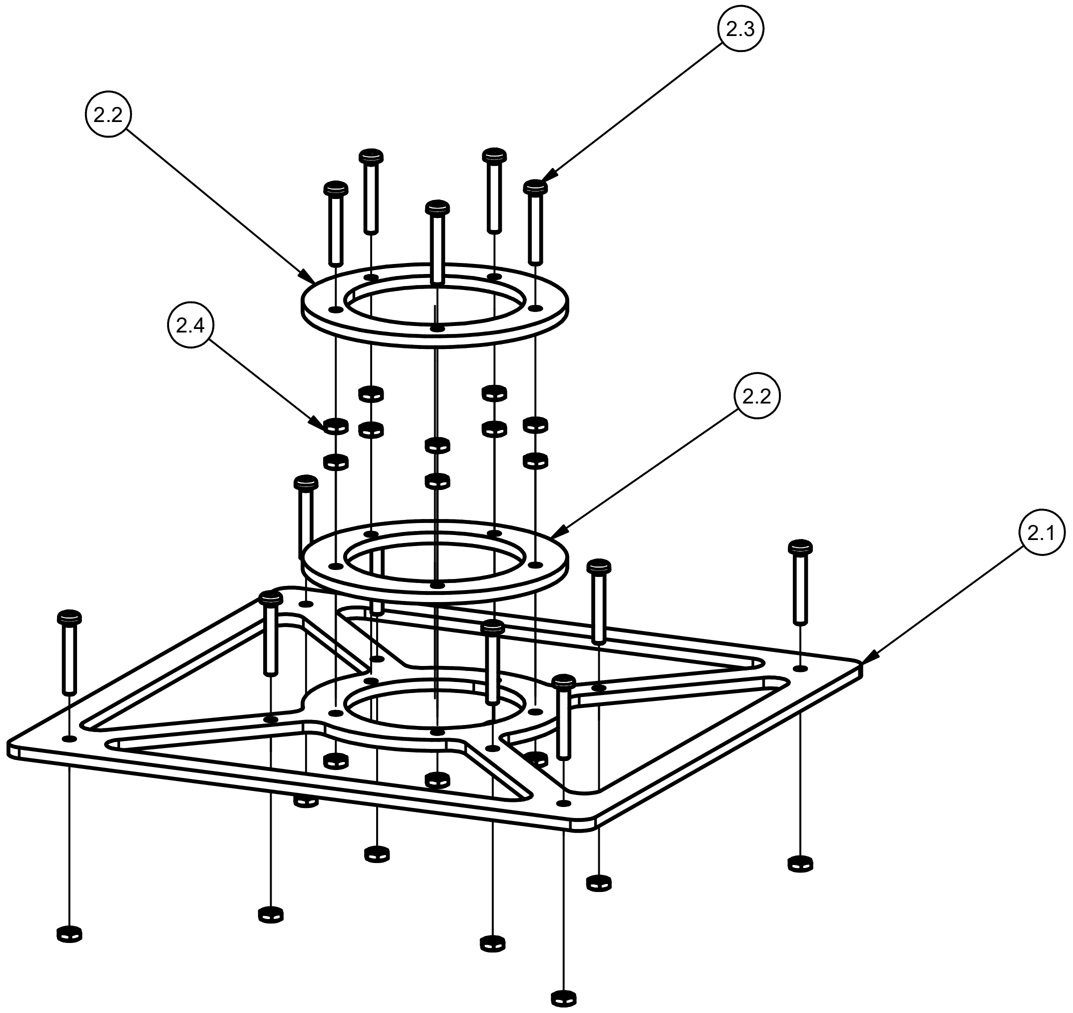

Item	Quantity	Part	Description
2.4	23	ISO 4032 - M3	Hex nuts
2.3	13	ISO 7045 - M3 x 20	Screws
2.2	2	Centering ring	3mm transparent PMMA
2.1	1	Support base	3mm transparent PMMA

	Name	Date
Designed	G. Salvador-Herranz	2020/06/22
Revised	R. Argilés	2020/06/22

Joint FAO/IAEA Programme

Insect Pest Control Section

Scale: mm

Flight Ability Test Device

Inner flight tube holder

Number: FATD_V1

Sheet: 6/11
