# Supplementary material for: Standardization of the FAO/IAEA Flight Test for Quality Control of Sterile Mosquitoes
Source: Front Bioeng Biotechnol. 2022 Jul 18;10:876675. doi: 10.3389/fbioe.2022.876675 (PMC9341283; doi:10.3389/fbioe.2022.876675)
Supplement: Supplementary file 1 [file DataSheet1.zip › Supplementary Materials/Supplementary Material S8. Inner tube_Flight Tube System Assembly.pdf]

3

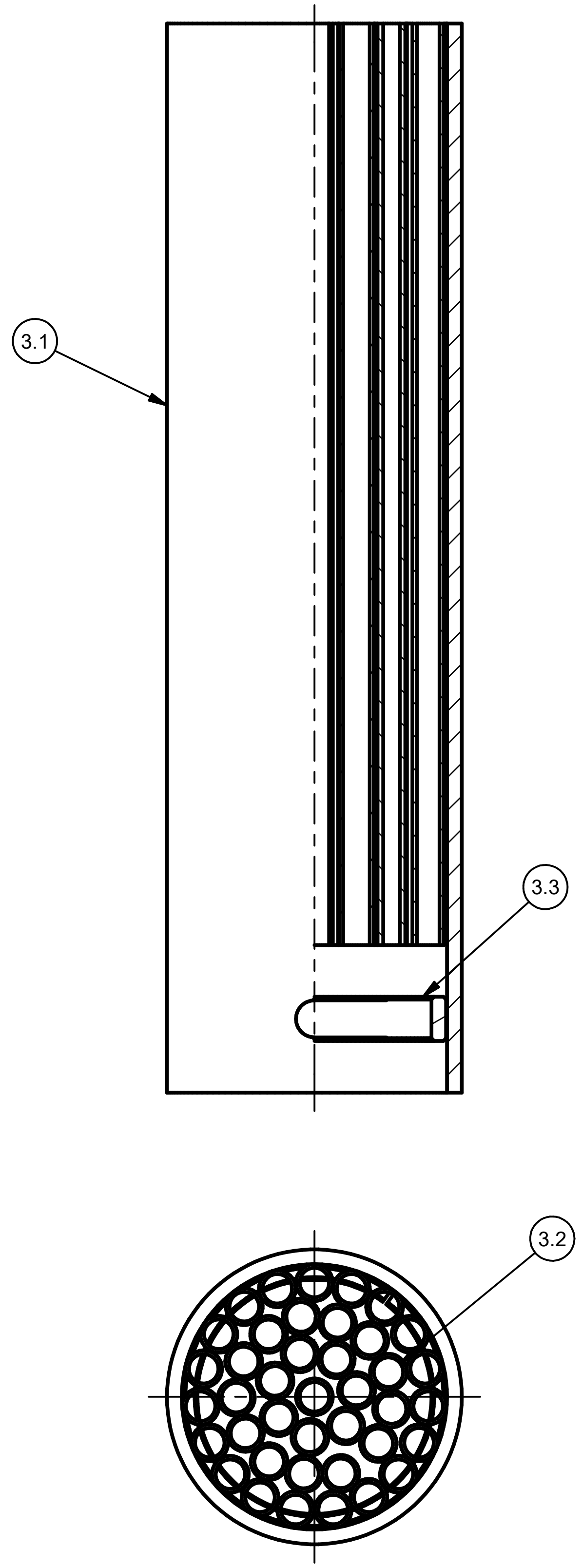

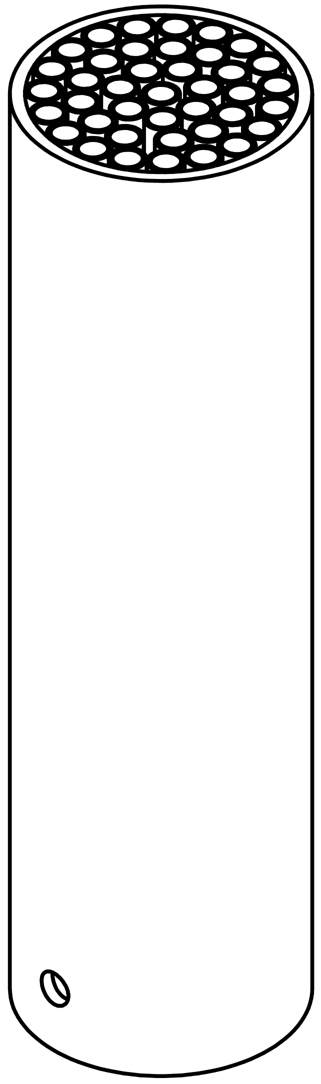

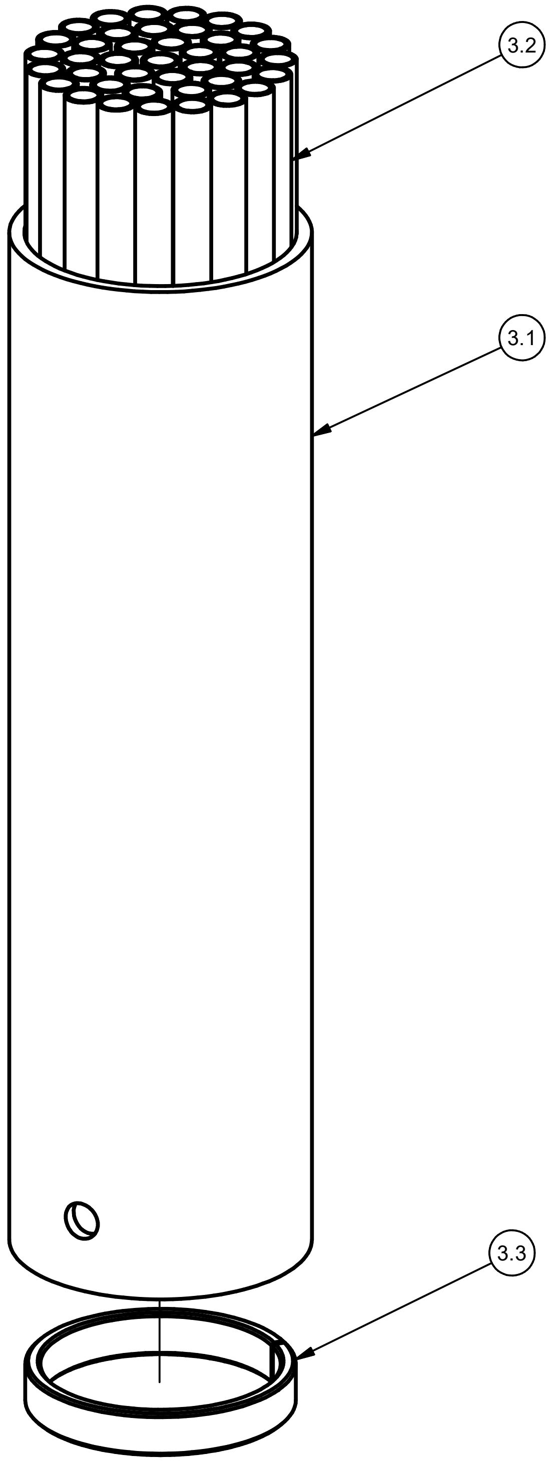

Item	Quantity	Part	Description
3.3	1	Mesh clamp ring	PVC
3.2	40	Tubos_Interiores	10x8mm transparent PMMA
3.1	1	Outer tube	4mm transparent PMMA

	Name	Date
Designed	G. Salvador-Herranz	2020/06/22
Revised	R. Argilés	2020/06/22

Joint FAO/IAEA Programme

Insect Pest Control Section

Scale		Number
mm	Flight Ability Test Device<br>Flight Tube System - Assembly	FATD_V1
		Sheet 8/11
