# Supplementary material for: Standardization of the FAO/IAEA Flight Test for Quality Control of Sterile Mosquitoes
Source: Front Bioeng Biotechnol. 2022 Jul 18;10:876675. doi: 10.3389/fbioe.2022.876675 (PMC9341283; doi:10.3389/fbioe.2022.876675)
Supplement: Supplementary file 1 [file DataSheet1.zip › Supplementary Materials/Supplementary Material S9. Inner Flight Tube Parts 1_1.pdf]

3.1

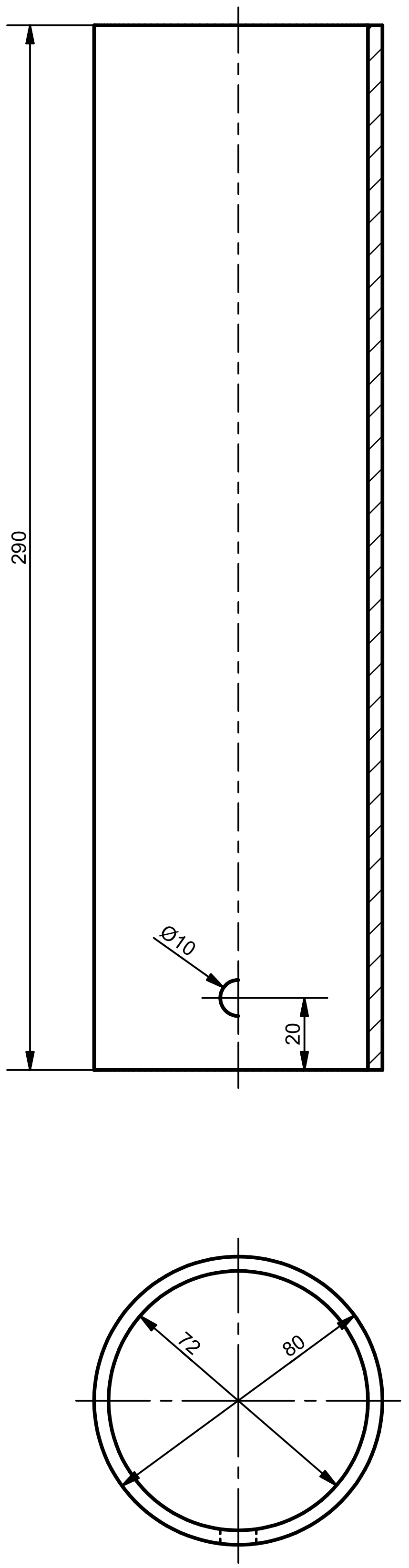

3.2

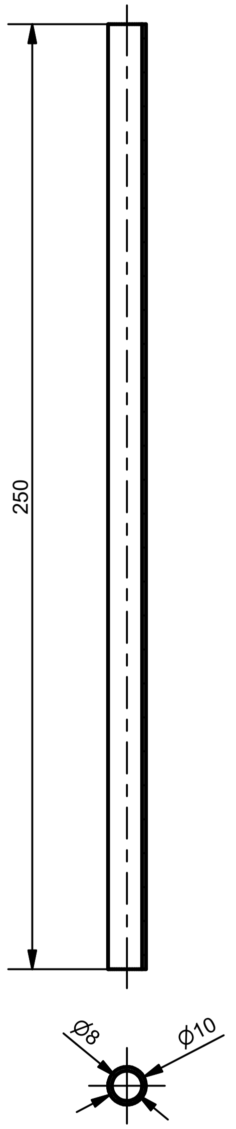

3.3

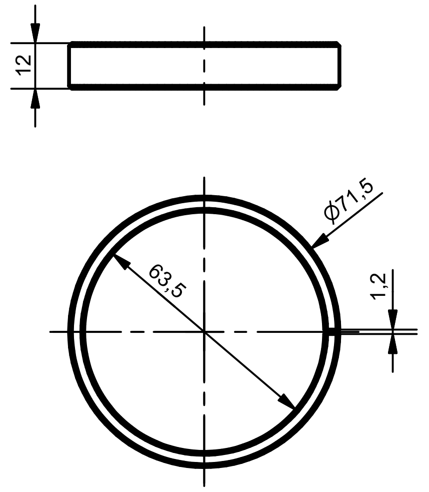

	Name	Date	Insect Pest Control Section
Designed	G. Salvador-Herranz	2020/06/22	
Revised	R. Argilés	2020/06/22	
Scale 1:1 mm	Flight Ability Test Device<br>Inner Flight Tube - Parts 1/1		Number FATD_V1<br>Sheet 9/11
