# Supplementary material for: Standardization of the FAO/IAEA Flight Test for Quality Control of Sterile Mosquitoes
Source: Front Bioeng Biotechnol. 2022 Jul 18;10:876675. doi: 10.3389/fbioe.2022.876675 (PMC9341283; doi:10.3389/fbioe.2022.876675)
Supplement: Supplementary file 1 [file DataSheet1.zip › Supplementary Materials/Supplementary Material S14_video.pptx]

## Slide 1
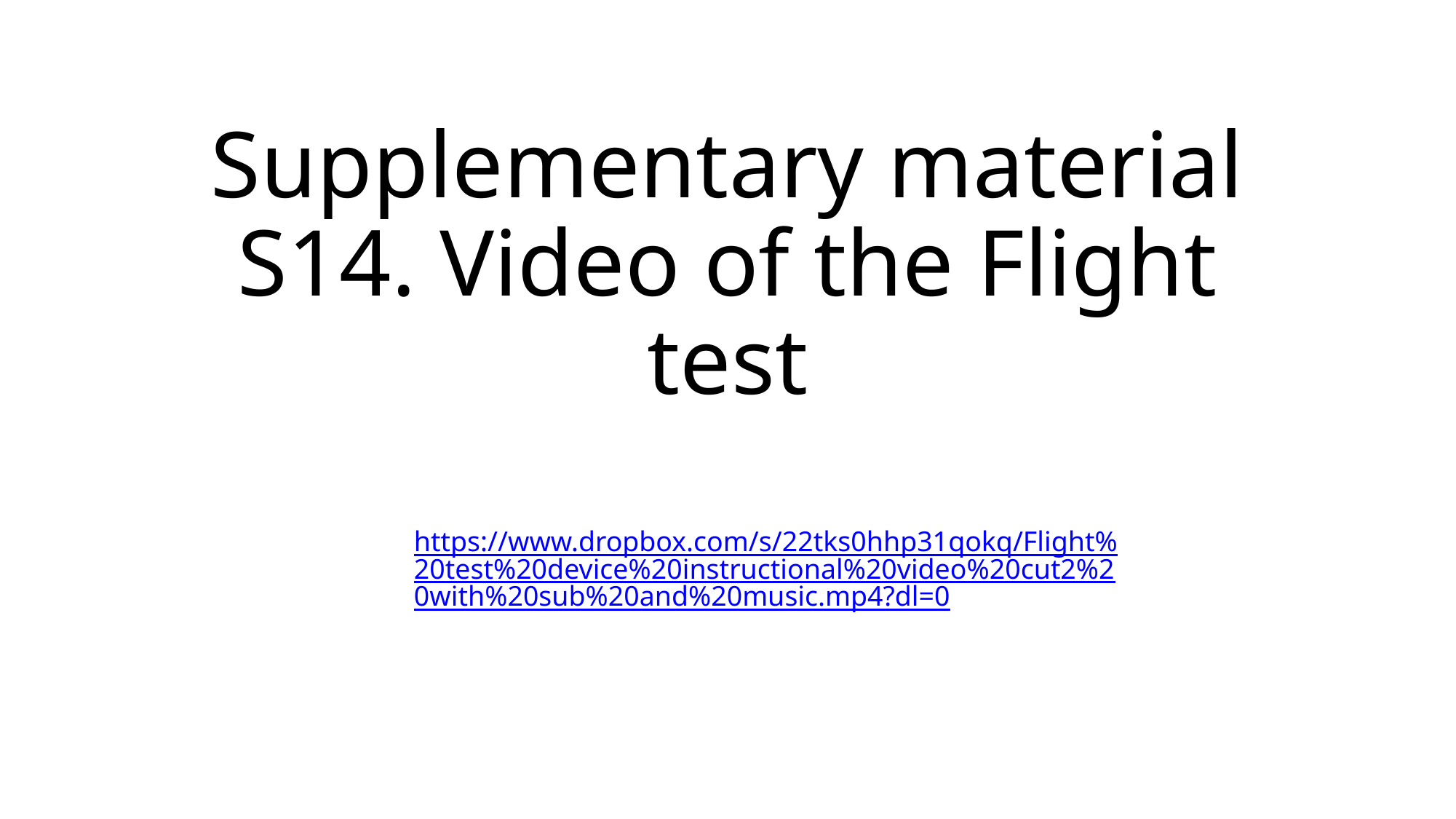

# Supplementary material S14. Video of the Flight test
https://www.dropbox.com/s/22tks0hhp31qokq/Flight%20test%20device%20instructional%20video%20cut2%20with%20sub%20and%20music.mp4?dl=0
