# Supplementary material for: Standardization of the FAO/IAEA Flight Test for Quality Control of Sterile Mosquitoes
Source: Front Bioeng Biotechnol. 2022 Jul 18;10:876675. doi: 10.3389/fbioe.2022.876675 (PMC9341283; doi:10.3389/fbioe.2022.876675)
Supplement: Supplementary file 1 [file DataSheet1.zip › Supplementary Materials/Supplementary Material S2. Overall View.pdf]

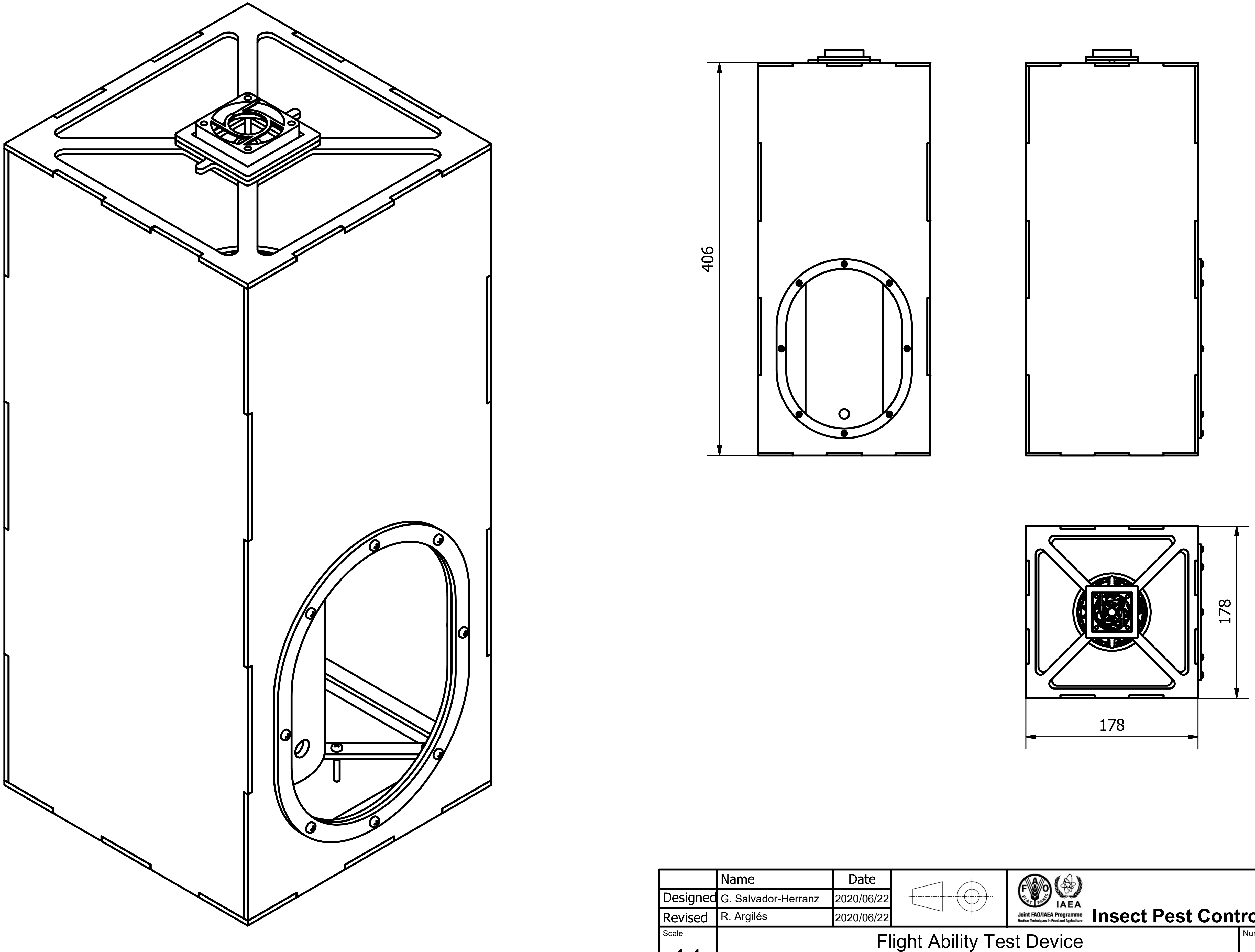

	Name	Date		
Designed	G. Salvador-Herranz	2020/06/22		Insect Pest Control Section
Revised	R. Argilés	2020/06/22		
Scale 1:4 mm	Flight Ability Test Device<br>Overall View			Number FATD_V1<br>Sheet 1/11
