# Supplementary material for: Standardization of the FAO/IAEA Flight Test for Quality Control of Sterile Mosquitoes
Source: Front Bioeng Biotechnol. 2022 Jul 18;10:876675. doi: 10.3389/fbioe.2022.876675 (PMC9341283; doi:10.3389/fbioe.2022.876675)
Supplement: Supplementary file 1 [file DataSheet1.zip › Supplementary Materials/Supplementary Material S3. Main Assembly.pdf]

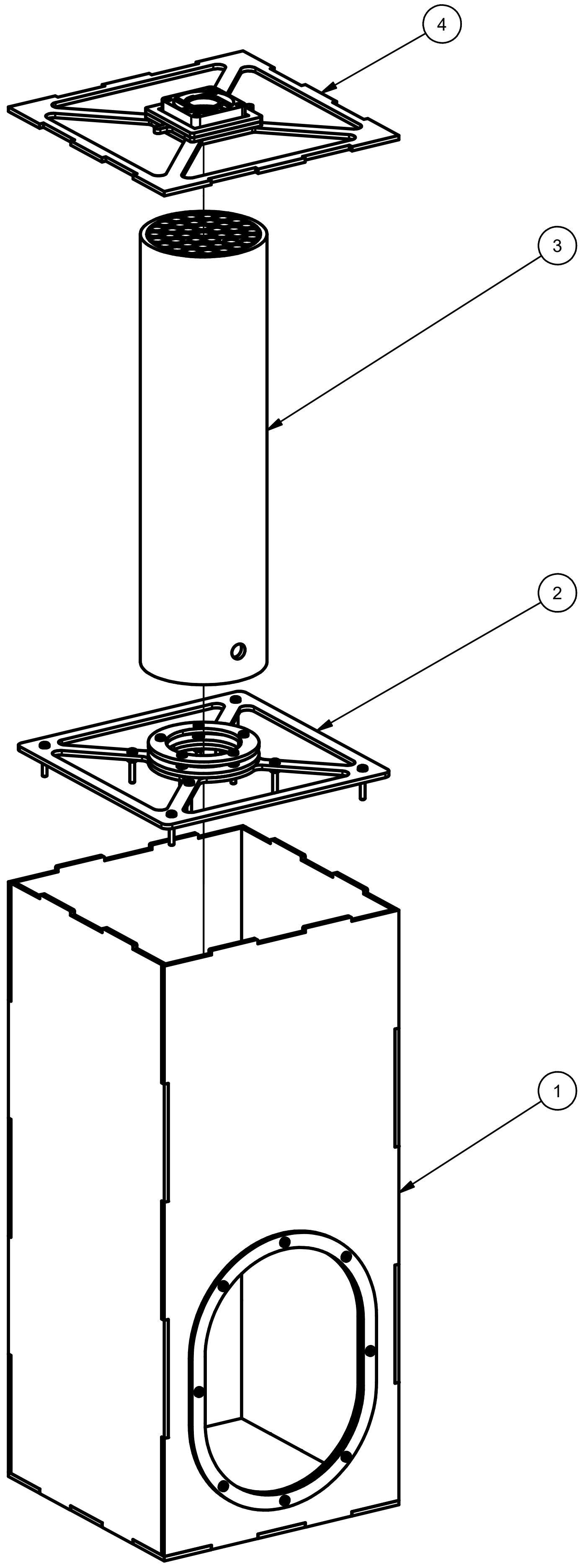

Item	Quantity	Part	Description
4	1	Top cover	
3	1	Flight tubes	
2	1	Inner tube holder	
1	1	Main container	

	Name	Date
Designed	G. Salvador-Herranz	2020/06/22
Revised	R. Argilés	2020/06/22

IAEA
Joint FAO/IAEA Programme
Nuclear Techniques in Food and Agriculture

Insect Pest Control Section

Scale: mm

Flight Ability Test Device

Main Assembly

Number: FATD_V1

Sheet: 2/11
