# Supplementary material for: Standardization of the FAO/IAEA Flight Test for Quality Control of Sterile Mosquitoes
Source: Front Bioeng Biotechnol. 2022 Jul 18;10:876675. doi: 10.3389/fbioe.2022.876675 (PMC9341283; doi:10.3389/fbioe.2022.876675)
Supplement: Supplementary file 1 [file DataSheet1.zip › Supplementary Materials/Supplementary Material S4. Outer Container Assembly.pdf]

# 1

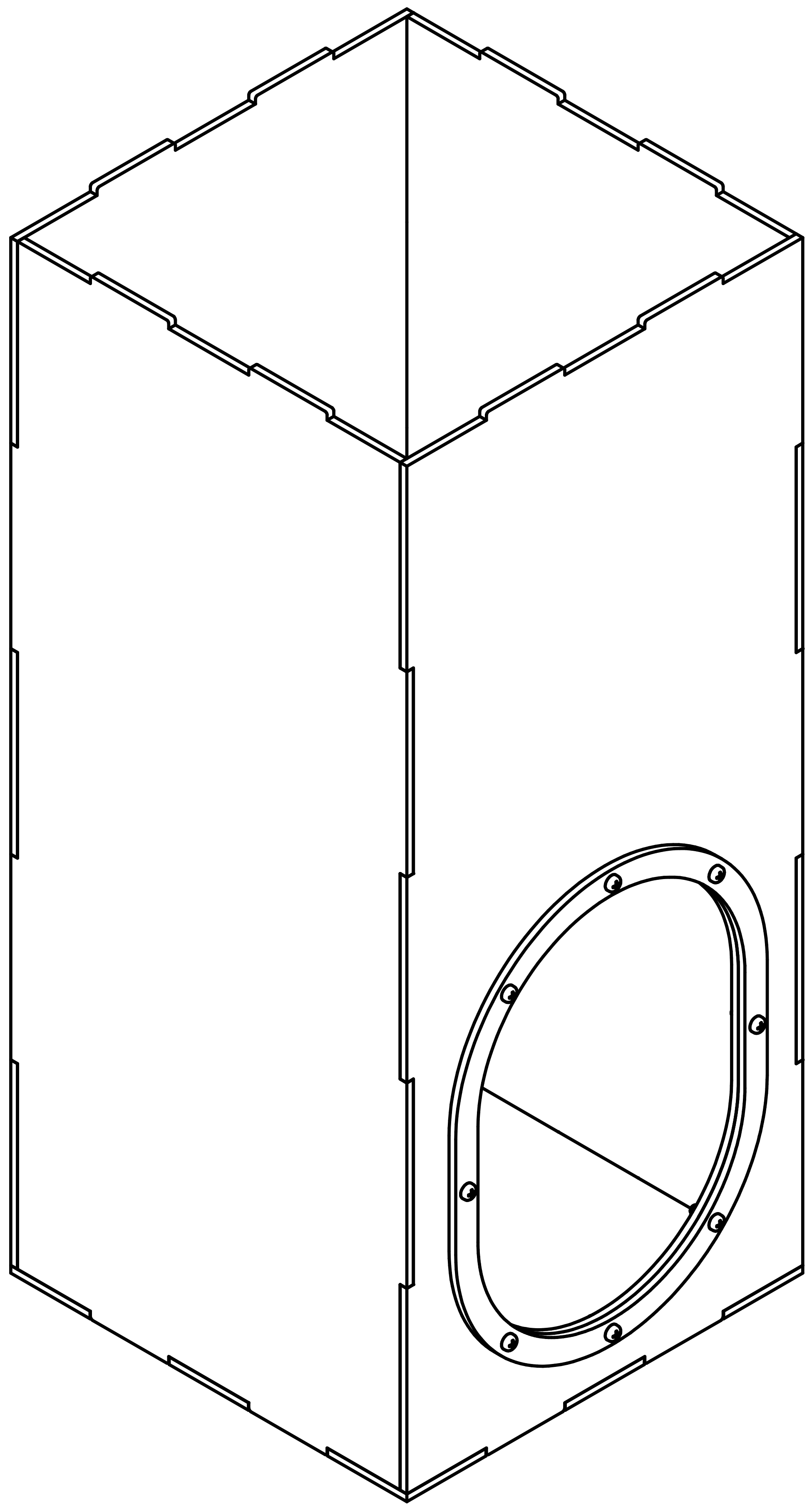

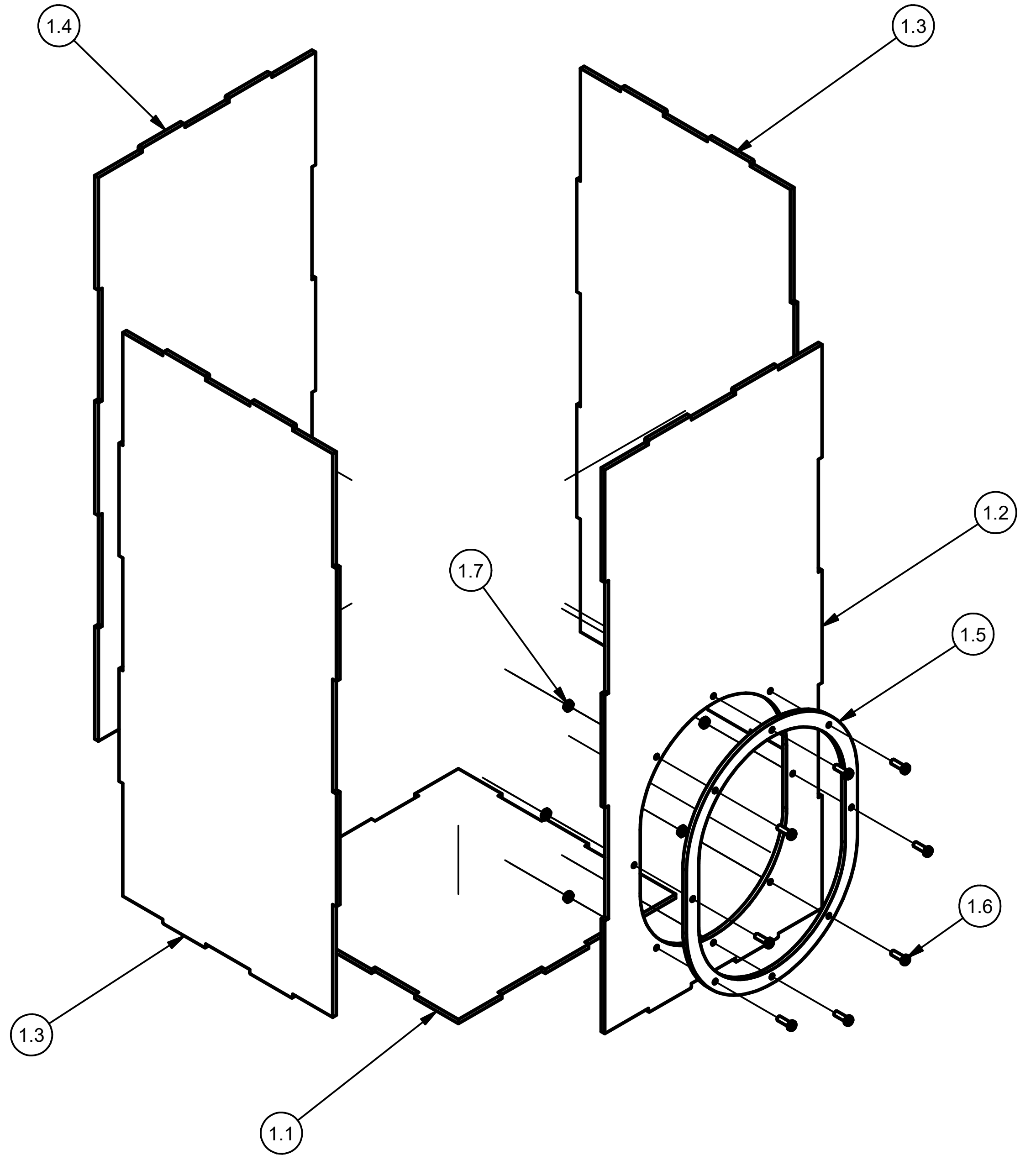


| Item | Quantity | Part | Description |
|---|---|---|---|
| 1.7 | 8 | ISO 4032 - M3 | Hex nuts |
| 1.6 | 8 | ISO 7045 - M3 x 10 | Screws |
| 1.5 | 1 | Sleeve retaining ring | 3mm transparent PMMA |
| 1.4 | 1 | Rear panel | 3mm transparent PMMA |
| 1.3 | 2 | Side panel | 3mm transparent PMMA |
| 1.2 | 1 | Front panel | 3mm transparent PMMA |
| 1.1 | 1 | Bottom base | 3mm transparent PMMA |

| | Name | Date |
|---|---|---|
| Designed | G. Salvador-Herranz | 2020/06/22 |
| Revised | R. Argilés | 2020/06/22 |

Joint FAO/IAEA Programme — IAEA

**Insect Pest Control Section**

Scale: mm

Flight Ability Test Device

Outer Container Assembly

Number: FATD_V1

Sheet: 3/11
