# Supplementary figures and images for: Standardization of the FAO/IAEA Flight Test for Quality Control of Sterile Mosquitoes
Source: Front Bioeng Biotechnol. 2022 Jul 18;10:876675. doi: 10.3389/fbioe.2022.876675 (PMC9341283; doi:10.3389/fbioe.2022.876675)

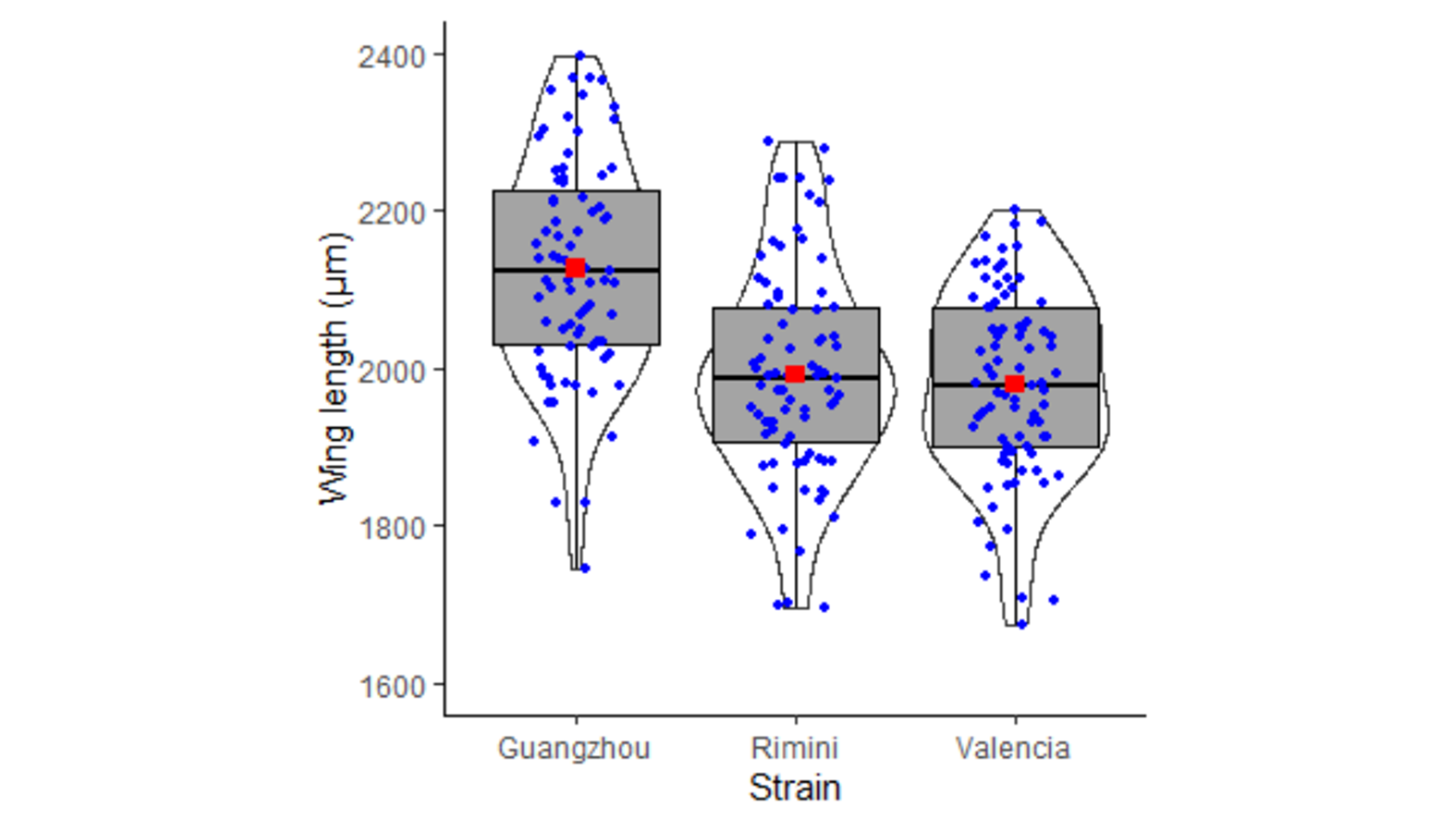

Supplement: Supplementary file 2 [file Image1.TIF]
